# Combination of a Sindbis-SARS-CoV-2 Spike Vaccine and αOX40 Antibody Elicits Protective Immunity Against SARS-CoV-2 Induced Disease and Potentiates Long-Term SARS-CoV-2-Specific Humoral and T-Cell Immunity

**DOI:** 10.3389/fimmu.2021.719077

**Published:** 2021-07-29

**Authors:** Antonella Scaglione, Silvana Opp, Alicia Hurtado, Ziyan Lin, Christine Pampeno, Maria G. Noval, Sara A. Thannickal, Kenneth A. Stapleford, Daniel Meruelo

**Affiliations:** ^1^Department of Pathology, New York University Grossman School of Medicine, New York, NY, United States; ^2^Department of Microbiology, New York University Grossman School of Medicine, New York, NY, United States

**Keywords:** Sindbis virus vaccine, αOX40, synergistic combination SARS-CoV-2 vaccine strategy, SARS-CoV-2 immunity, alphavirus vaccine, COVID19, SARS-CoV-2 vaccine

## Abstract

The COVID-19 pandemic caused by the coronavirus SARS-CoV-2 is a major global public threat. Currently, a worldwide effort has been mounted to generate billions of effective SARS-CoV-2 vaccine doses to immunize the world’s population at record speeds. However, there is still a demand for alternative effective vaccines that rapidly confer long-term protection and rely upon cost-effective, easily scaled-up manufacturing. Here, we present a Sindbis alphavirus vector (SV), transiently expressing the SARS-CoV-2 spike protein (SV.Spike), combined with the OX40 immunostimulatory antibody (αOX40) as a novel, highly effective vaccine approach. We show that SV.Spike plus αOX40 elicits long-lasting neutralizing antibodies and a vigorous T-cell response in mice. Protein binding, immunohistochemical, and cellular infection assays all show that vaccinated mice sera inhibits spike functions. Immunophenotyping, RNA Seq transcriptome profiles, and metabolic analysis indicate a reprogramming of T cells in vaccinated mice. Activated T cells were found to mobilize to lung tissue. Most importantly, SV.Spike plus αOX40 provided robust immune protection against infection with authentic coronavirus in transgenic mice expressing the human ACE2 receptor (hACE2-Tg). Finally, our immunization strategy induced strong effector memory response, potentiating protective immunity against re-exposure to SARS-CoV-2 spike protein. Our results show the potential of a new Sindbis virus-based vaccine platform to counteract waning immune response, which can be used as a new candidate to combat SARS-CoV-2. Given the T-cell responses elicited, our vaccine is likely to be effective against variants that are proving challenging, as well as serve as a platform to develop a broader spectrum pancoronavirus vaccine. Similarly, the vaccine approach is likely to be applicable to other pathogens.

## Introduction

In the ongoing COVID19 pandemic, vaccines play a key role in the strategy to bring SARS-CoV-2 transmission under control. Safety and eliciting a broad-spectrum immune response are paramount for coronavirus vaccine development. Data from vaccine clinical trials and real-world evidence show that available coronavirus vaccines are able to cut the risk of severe COVID19 disease and transmission. However, even with first-generation vaccines currently being globally administered to reduce transmission and severity of the disease, the emergence of circulating variants has raised major concerns that challenge sustained vaccine efficacy, particularly in the face of waning immunity following vaccination (1–7). Recent data have indicated that escape (appearance and spread of viral variants that can infect and cause illness in vaccinated hosts) from protection by vaccines designed against the Wuhan-1 strain is inevitable (4).

The global COVID19 pandemic is unlikely to end until there is an efficient pan-global roll-out of SARS-CoV-2 vaccines. Although multiple vaccines are currently available, vaccine rollout and distribution at the time of writing this paper is quite incomplete. The three largest countries in the western hemisphere—United States, Brazil, and Mexico—have vaccinated 46%, 13%, and 15% of their populations, respectively, compared with only 4.2% in India (8). Vaccine distribution to date has been highly non-uniform among these and other countries around the globe, encountering many challenges. Unequal vaccine roll-out and the new B.1.617 variant are highly concerning. Major challenges have been supply shortages, logistic problems, complex storage conditions, priced affordably, and safety (9). Consequently, the pandemic is currently sweeping through India at a pace faster than ever before. The countries’ second wave became the worst COVID19 surge in the world, despite previous high infection rates in megacities that should have resulted in some immunity. More cost-effective and facilitated delivery of broad-spectrum SARS-CoV-2 vaccines would help improve wide and rapid distribution that would in turn minimize vaccine escape.

Traditionally, vaccines have been designed to induce antibody responses and have been licensed on their capacity to induce high titers of circulating antibody to the pathogen (10). With increased knowledge of host-virus interactions, it has become clear that the cellular arm of the immune response is also crucial for the efficacy of vaccines against pathogens and to provide appropriate help for antibody induction. Various strategies have emerged that specialize in developing candidate vaccines that solely induce either cellular or humoral responses (10). However, as most viruses and pathogens reside at some point during their infectious cycle in the extracellular, as well as intracellular space, vaccines need to promptly elicit a strong T-cell memory response against intracellular pathogens, so that, at the earliest stages of the infective process, prevention can be addressed in coordination with antibodies.

It has been reported that recovered COVID19 patients consistently generate a substantial CD4+ T (OX40+CD137+) cell response against SARS-CoV-2 spike (11). SARS-CoV-2−specific CD4+ T cells produced IL-2 and substantial amounts of IFN*γ*, hallmarks of Th-1 type effector T-cell polarization. Th-1 type effector T cells provide critical help for CD8 T-cell priming and conferring cytotoxic T cell–mediated immune protection. The costimulatory molecule OX40 is a member of TNF receptor superfamily (TNFRSF) that is upregulated on activated T cells shortly after T-cell receptor recognition of specific antigen (12, 13). It is mainly expressed on CD4+ T cells, although activated CD8 T also express OX40, albeit at lower levels (14). Once activated, OX40 receptor is the key molecule for clonal expansion, differentiation, and survival of Th1-effector cells and cytokine production (12, 15–19). Although OX40 does not directly initiate T-cell memory formation, it contributes to homeostasis of memory T cells and enhances effector memory T-cell function (20). In addition to its role in direct T cell–mediated viral clearance (T-cell immunity), OX40 stimulation is found to cooperate with the inducible costimulating (ICOS) molecule on follicular T helper (Tfh) cells augmenting their amplification and development to coordinate humoral immune response (21). Antigen-specific activated Tfh cells help B cells produce high-affinity antibodies against pathogens and are indispensable for vaccine-induced long-lasting humoral immunity by facilitating differentiation of memory B cells and long-lived plasma cells from Germinal Centers (GC) (22–24). Therefore, designing a vaccine that could stimulate OX40 would provide a powerful platform for T cell–mediated immunity.

Alphaviruses have demonstrated strong attributes as a development-and-manufacturing platform for vaccines (25–32). Particularly, studies with SARS-CoV strains bearing epidemic and zoonotic spike variants are promising (31). The strength of the use of alphavirus vaccine utilization is the generation of rapid, high-level, and transient nature of transgene expression (33). Importantly, we have shown in our earlier preclinical work (34–36) that alphavirus vaccine platforms have the advantage to directly deliver antigens and immune modulatory molecules to lymph nodes, where they are expressed transiently to elicit diversified CD4+ and CD8+ T-cell immunity effective at controlling tumors throughout the body. These vectors represent a highly effective self-amplifying mRNA vaccine that can be engineered to express multiple antigens and stimulatory molecules. Within 3 h after infection, the vector generates hundreds of thousands of mRNA copies within the infected cells and high levels transgene expression (e.g., the spike antigen and anti-OX40 antibody) (37). At the same time, the transient nature and cytosolic location of RNA improve the safety profile of SV vector-based vaccines. The replication defective nature of our vectors ensures no further transmission of the virus beyond the infected cells (38). Replication-deficient alphavirus-based vaccines are immunogenic, safe, well tolerated, and can be cost-effectively stored and transported using conventional 2°C to 8°C storage as well as lyophilization (39, 40).

Here we describe a new Sindbis virus (SV) vaccine transiently expressing the SARS-CoV-2 spike protein (SV.Spike). Adaptive immunity induced by SV.Spike fully protects transgenic mice that express the SARS-CoV receptor (human angiotensin-converting enzyme 2 [hACE2]), hACE2-Tg, against authentic SARS-CoV-2 virus infection. In addition, we demonstrate that combination of our vaccine with αOX40 agonistic antibody significantly enhances the induction of immunity by the SV.spike vector. Specifically, seroconversion and abundance of IgG neutralizing antibodies and T-cell immunity through early initiation of Th1-type T-cell polarization are markedly augmented to potentiate long-term immunity protective against SARS-CoV-2 infection in mice. Together these studies develop a safe and effective vaccine platform that provides humoral and cellular immunity to the SARS-CoV-2 spike. This platform has the potential application to other emerging pathogens.

## Materials and Methods

### Cell Lines

Baby hamster kidney (BHK) and 293T-cell lines were obtained from the American Type Culture Collection (ATCC). 293T/ACE2 cell line was obtained from BEI Resources.

BHK cells were maintained in minimum essential α-modified media (α-MEM) (Corning CellGro) with 5% fetal bovine serum (FBS, HyClone) and 100 mg/ml penicillin-streptomycin (Corning CellGro). 293T and 293T/ACE2 cells were maintained in Dulbecco’s modified Eagle’s medium containing 4.5 g/L Glucose (DMEM, Corning CellGro) supplemented with 10% FBS, 100 mg/ml penicillin-streptomycin. All cell lines were cultured at 37°C and 5% CO_2_.

### SV Production

SV.Spike expressing vector was produced as previously described (34, 35, 41, 42). Briefly, plasmids carrying the replicon (pT7-SV-Spike) or the helper T7-DMHelper RNAs were linearized with XhoI. *In vitro* transcription was performed using the mMessage mMachine RNA transcription kit (Invitrogen Life Sciences). Helper and replicon RNAs were then electroporated into BHK cells and incubated at 37°C in αMEM supplemented with 10% FBS. After 12 h, the media was replaced with OPTI-MEM (GIBCO-BRL) supplemented with CaCl_2_ (100 mg/l) and cells were incubated at 37°C. After 24 h, the supernatant was collected, centrifuged to remove cellular debris, and frozen at −80°C. Vectors were titrated as previously described (43) and titer expressed as transducing units per milliliter (TU/ml).

### Pseudotyped Lentivirus Production

SARS CoV-2 pseudotyped lentiviruses were produced by transfecting the 293T cells with the pLenti-Puro vectors (Addgene) expressing Luciferase or β-Galactosidase, with pcDNa3.1 vector expressing SARS-CoV-2 spike (BEI repository) and the helper plasmid pSPAX2 (Addgene). The VSV-G and empty lentiviruses were produced by replacing pCDNA3.1-Spike with pcDNA3.1-VSV-G or pCDNA3.1 empty vector, respectively (Addgene). The transfections were carried out using the polyethylenimine (PEI) method with the ratio at PEI:pLenti:pcNDA3.1-Spike:pSPAX2 = 14:2:2:1 or PEI:pLenti:pVSV-G/pcNDA3.1:pSPAX2 = 10:1:0.5:3. The virus-containing medium was harvested 72 h after transfection and subsequently pre-cleaned by centrifugation (3,000*g* at 4°C) and a 0.45 μm filtration (Millipore). The virus-containing medium was concentrated by using a LentiX solution (TakaraBio) a 10:1 v/v ratio and centrifuged at the indicated RCF at 4°C for 45 min. After centrifugation, the supernatant was carefully removed, and the tube was drained on the tissue paper for 3 min. Dulbecco’s modified Eagle’s medium containing 4.5 g/L glucose (DMEM) was added to the semi-dried tube for re-suspension and then stored at −80°C.

### Detection of SARS-CoV-2 Spike Pseudotyped Lentivirus Infectivity

*Luciferase*- and *nLacZ*-encoding SARS CoV-2 Spike or VSV-G pseudotyped lentivirus titers were determined making serial dilutions of the vectors in DMEM and by infecting 293T/ACE2 cells pre-plated in 96-well culture plates (10^4^ cells/well). 24 h later, fresh media was added. For *Luciferase*-encoding pseudotype, cells were lysed 72 h later using cell lysis buffer and lysates were transferred into fresh 96-well luminometer plates, where luciferase substrate was added (Thermo Fisher), and relative luciferase activity was determined (**Supplementary Figure 4C**). For *nLacZ*-encoding pseudotypes, cells were washed with PBS and stained for 16 h at 37°C with X-Gal Solution [1 mg/ml X-Gal in PBS (pH 7. 0) containing 20 mM potassium ferricyanide, 20 mM potassium ferrocyanide, and 1mM MgCl2] (**Supplementary Figure 4D**). Vector titers refer to the number of infectious particles (transducing units per milliliter of supernatant [TU/mL] and were estimated as the last dilution having detectable reporter activity. Correct assembling of pseudotypes was assessed by western blot following standard protocol, to detect the expression of SARS-CoV-2-spike and p24 proteins. SARS-CoV-2 spike (BPS Bioscience) and p24 (Abcam) recombinant proteins were used as positive controls (**Supplementary Figures 4A, B**).

### *In Vivo* Experiments

All experiments were performed in accordance with protocols approved by the Institutional Animal Care and Use Committee of New York University Grossman School of Medicine. Six- to 12-week-old female C57BL/6J albino mice (B6(Cg)-Tyr<c-2J>/J,Cat 000058) and hemizygous (B6(Cg)-Tg(K18-ACE2)2Prlmn/J; Cat 034860) (hACE2-Tg) mice expressing the human ACE2 receptor or non-carrier controls were purchased from Jackson Laboratory.

### ABSL3 Experiments Using SARS-CoV-2 Coronavirus

Three weeks after prime and boost vaccination doses, hACE2-Tg and non-carrier control mice were challenged with 10^4^ pfu particles of SARS-CoV-2 Coronavirus *via* the intranasal (i.n.) route (**Figure 4F**). We recorded daily the body weight of each mouse after infection for a total of 14 days. The New York University Grossman School of Medicine (NYUSOM) Animal Biosafety Level 3 (ABSL3) Facility, located on the third floor of the Alexandria Center for Life Science West Tower, is a 3,000 sq. ft. high-containment research facility under the responsibility of the Office of Science & Research and its Director of High-Containment Laboratories. It has been designed, and it is operated in compliance with the guidelines of the Centers for Disease Control and Prevention (CDC) and the National Institutes of Health (NIH). All research and non-research operations are governed by institutional standard operating procedures (SOPs). As per those SOPs, all users undergo specific training and require medical and respiratory protection clearance. The facility and its SOPs are re-certified by an outside consultant on a yearly basis. The NYUSOM ABSL3 has also been registered with the Department of Health and Mental Hygiene of the city of New York since March 2017.

Exposure of the animals to pseudotyped (**Figures 4D, E** **)**, or authentic SARS-CoV-2 (**Figures 4F–H**) or SV.Spike only (**Figure 11**) subsequent to prime and boost vaccination doses is defined in the manuscript as *challenge* or *recall*. Both terms will be mostly interchangeable, where the term challenge describes the condition of the animals after exposure to the authentic coronavirus, whereas recall or “challenge” will be used for reference to the ability of the antigen from pseudotyped SARS-CoV-2 and SV-Spike to recall the immune response.

### Mouse Vaccination and Serum Collection

Mice were intraperitoneally (i.p.) immunized with SV.Spike (10^7^ TU/ml) in a total volume of 500 μl was injected i.p. into the left side of the animal. The immunostimulatory αOX40 antibody (clone OX-86, BioXCell) was injected i.p. into the left side of the animal at a dose of 250 μg per injection. Mice were boosted once at 2 weeks. Sera were collected at 7 days post-2nd vaccination and used to detect neutralizing antibody (NAbs) activity.

Therapeutic efficacy of vaccines was monitored in two ways: vaccinated hACE2-Tg mice that were challenged with SARS-CoV-2 coronavirus in BSL3 were tested for survival compared to their non immunized control group. Survival and weight was monitored and recorded daily.

### *In Vivo* Delivery of nLacZ-SARS-CoV-2 Pseudotyped Lentivirus and X-Gal Histochemistry

Isoflurane-anesthetized 4-week-old young adult hACE2-Tg mice were dosed intranasally with a 70-µl volume of *nLacZ*-encoding lentiviral vector (titer 5.18 × 10^3^ TU/ml). Isoflurane anesthesia (2.5% isoflurane/1.5 L oxygen per minute), and dosing of animals was carried out in a vented BSL-2 biological safety cabinet. For processing of mouse lungs for X-Gal staining of intact tissue, lungs were inflated through the trachea with OCT embedding as described previously (44). Intact airways were submerged in 0.5% glutaraldehyde for 2 h at 4°C, washed in PBS/1 mM MgCl_2_ and stained for 16 h at 37°C with X-Gal Solution [1 mg/ml X-Gal in PBS (pH 7. 0) containing 20 mM potassium ferricyanide, 20 mM potassium ferrocyanide, and 1mM MgCl_2_].

### Neutralization Experiments

#### SARS-CoV-2 Spike-hACE2 Blocking Assay

To measure protective NAbs, COVID-19 convalescent plasma from C57BL/6J mice was diluted (1:10) and incubated with recombinant SARS-CoV-2 full-length Spike (BPS Bioscience) for 1 h at 37°C prior to adding to an hACE2 pre-coated ELISA plates. The NAb levels were calculated based on their inhibition extents of Spike and hACE2 interactions according to the following equation: [(1-OD value of samples/OD value of negative control) × 100%]. A neutralizing antibody against SARS-CoV-2 spike (Bio Legend) was used as a positive control.

#### SARS-CoV-2 Spike Pseudotyped Lentivirus Inhibition Assay

Pseudotyped lentivirus inhibition assay was established to detect neutralizing activity of vaccinated mouse sera and inhibitory ability of antiviral agents against infection of SARS-CoV-2 spike pseudotyped lentivirus in target cells. Briefly, pseudotyped expressing luciferase virus containing supernatants were respectively incubated with serially diluted mouse sera at 37°C for 1 h before adding to target cells pre-plated in 96-well culture plates (10^4^ cells/well). 24 h later, fresh media was added, and cells were lysed 72 h later using cell lysis buffer. Lysates were transferred into fresh 96-well luminometer plates. Luciferase substrate was added (Promega), and relative luciferase activity was determined. The inhibition of SARS-COV-2 Spike pseudotype lentivirus was presented as % inhibition.

### Cell–Cell Fusion Assay

The establishment and detection of several cell–cell fusion assays are as previously described (45). In brief, 293T/ACE2 cells were used as target cells. For preparing effector cells expressing SARS-CoV-2 spike, 293T cells were transiently co-transfected with pCDNA3.1-Spike and pMAX-GFP or with pMAX-GFP only as control, and applied onto 293T/ACE2 cells after 48 h from transfection. Effector and target cells were cocultured in DMEM plus 10% FBS for 6 h. After incubation, five fields were randomly selected in each well to count the number of fused and unfused cells under an inverted fluorescence microscope (Nikon Eclipse Ti-S).

### Inhibition of SARS-CoV-2-Spike-Mediated Cell–Cell Fusion

The inhibitory activity of neutralizing antibodies from immunized mice sera on a SARS-CoV-2-spike-mediated cell–cell fusion was assessed as previously described (46, 47).

Briefly, a total of 2 × 10^4^ target cells/well (293T/ACE2) were incubated for 5 h. Afterward, medium was removed, and 10^4^ effector cells/well (293T/Spike/GFP) were added in the presence of serum from C57BL/6J immunized mice at 1:100 dilution in medium at 37°C for 2 h. The fusion rate was calculated by observing the fused and unfused cells using fluorescence microscopy.

### Immunocytochemistry

Cell immunocytochemistry was performed as described previously (48). Briefly, cells were fixed with 4% paraformaldehyde (PFA) for 20 min at room temperature, and then the membrane was permeabilized with 0.1% (vol/vol) Triton X-100 (Fisher Scientific). Incubation with blocking solution (5% normal goat serum) was performed at room temperature for 45 min. Anti-mouse SARS-CoV-2-spike (GTX, 1:100) and anti-rabbit hACE2 (Thermo Fisher,1:100) were applied overnight at 4°C followed by incubation of appropriate secondary antibodies conjugated with fluorophores. Confocal images were captured using the Zeiss LSM-800 system.

### Flow Cytometry

For flow cytometry analysis, spleens were harvested from mice and processed as previously described (35). Extracted lungs were chopped in small pieces and incubated with a digestive mix containing RPMI with collagenase IV (50 μg/ml) and DNAseI (20 U/ml) for 30 min at 37°C. Spleens and lungs were mashed through a 70-μm strainer before red blood cells were lysed using ammonium-chloride-potassium (ACK) lysis buffer (Gibco). Cells were washed with PBS containing 1% FBS and surface receptors were stained using various antibodies. Fluorochrome-conjugated antibodies against mouse CD3, CD4, CD44, CD38, ICOS, OX40, CD62L, Perforin, Granzyme B, and Tbet, CXCR5 were purchased from Biolegend. Fluorochrome-conjugated antibodies against mouse CD8a were purchased from BD Biosciences. Fluorochrome-conjugated antibodies against CXCR3 and Ki67 were purchased from Thermofisher. Stained cells were fixed with PBS containing 4% Formaldehyde. For intracellular staining, the forkhead box P3 (FOXP3) staining buffer set was used (eBioscience). Flow cytometry analysis was performed on a LSR II machine (BD Bioscience) and data were analyzed using FlowJo (Tree Star).

### T- and B-Cell Isolation

Total T cells were freshly isolated with the EasySep™ mouse T Cell Isolation Kit. Total B cells were freshly isolated with the EasySep™ mouse B-cell Isolation Kit. Isolation of T and B cells were performed according to the manufacturer’s protocols (Stemcell Technologies).

### Enzyme-Linked Immunospot

Enzyme-linked immunospot was performed as previously described (35). Mouse IFNγ ELISPOT was performed according to the manufacturer’s protocol (BD Bioscience). Freshly isolated T cells (1 × 10^5^/well) were directly plated per well overnight in RPMI supplemented with 10% FBS. No *in vitro* activation step was included. As positive control, cells were stimulated with 5ng/ml PMA+1μg/ml Ionomycin.

### *Ex Vivo* Cytotoxic Assay

T cells (8 × 10^5^/ml) from C57BL/6J immunized splenocytes were co-cultured with 293T/ACE2 cells (2 × 10^4^/ml), previously infected with 3 × 10^5^ TU of SARS-CoV-2 Luc-SARS-CoV-2 spike pseudotyped lentivirus. Cells were co-cultured in a 24-well plate for 2 days in 1 ml of RPMI 1640 supplemented with 10% FBS, washed with PBS, and lysed with 100 μl of M-PER mammalian protein extraction reagent (Thermo Fisher) per well. Cytotoxicity was assessed based on the viability of 293T/ACE2 cells, which was determined by measuring the luciferase activity in each well. Luciferase activity was measured by adding 100 μl of Steady-Glo reagent (Promega) to each cell lysate and measuring the luminescence using a GloMax portable luminometer (Promega).

### Transcriptome Analysis of T Cells

Total RNA was extracted from freshly isolated T cells on day 7 of treatment from spleens using RNeasy Kit (Qiagen). For each group, five C57BL/6J mice were used for biological repeats. RNA-seq was done by NYUMC Genome Center. RNA quality and quantity were analyzed. RNAseq libraries were prepared and loaded on the automated Illumina Novae 6000 Sequencing System (Illumina). 1× S1 100 Cycle Flow Cell v1.5, 30 automated stranded RNA-seq library prep polyA selection, per sample.

### RNA-Seq Data Analysis

RNA-seq data were analyzed by sns rna-star pipeline (https://github.com/igordot/sns/blob/master/routes/rna-star.md). Sequencing reads were mapped to the reference genome (mm10) using the STAR aligner (v2.6.1d) (49). Alignments were guided by a Gene Transfer Format (GTF) file. The mean read insert sizes and their standard deviations were calculated using Picard tools (v.2.18.20) (http://broadinstitute.github.io/picard). The read count tables were generated using subread (v1.6.3) (50), (normalized based on their library size factors using DEseq2 (51), and differential expression analysis was performed. To compare the level of similarity among the samples and their replicates, we used principal-component. All the downstream statistical analyses and generating plots were performed in R environment (v4.0.3) (https://www.r-project.org/). The results of gene set enrichment analysis were generated by GSEA software (52, 53). The network of Gene Ontology terms was generated by Enrichment Map in Cytoscape. Additional protein–protein functional associations used in this study for bar graphs were retrieved from STRING (http://www.string-db.org/, version 11) (54), a well-known public database on several collected associations between proteins from various organisms.

### Measurement of Oxygen Consumption and Extracellular Acidification Rates of T and B Cells

T- and B-cell metabolic output was measured by Seahorse technology as previously described (55). Purified T cells from C57BL/6J immunized or control mice were plated at 6 × 10^5^ cells/well in a Seahorse XF24 cell culture microplate. Oxygen consumption rate (OCR) and extracellular acidification rate (ECAR) were measured using an Agilent Seahorse XFe24 metabolic analyzer following the procedure recommended by the manufacturer (Agilent). For the mitochondrial stress test: (1) oligomycin (1 μM), (2) FCCP (1.5 μM), and (3) rotenone (100 nM) and antimycin A (1 μM) were injected sequentially through ports A, B, and C.

### Immunoblot Analysis

Western blot was performed to detect SARS-CoV-2 spike protein in 293T cells infected with SV.Spike and in the generated pseudotyped lentivirus. Cells were lysed in M-PER^®^ Mammalian Protein Extraction Reagent (Thermo Fisher) according to the manufacturer’s protocol. Lysates were separated by SDS-PAGE on 4-12% Bio-Rad gels, transferred to polyvinylidene difluoride (PVDF) membranes, blocked in 5% milk in TBS buffer with 0.1% Tween-20 (TBST). Primary antibodies to SARS-CoV-2 spike (GTX, 1:1000) and p24 (Abcam, 1:1000) were added overnight at 4°C. HRP-conjugated secondary antibodies were added in 5% milk in TBST for 1 h at room temperature. BioRad Imaging System was used for visualization.

### Statistical Analysis

Statistical analysis was performed using GraphPad Prism 7.0 as described in figure legends. All data are shown as mean ± SEM. Figures were prepared using GraphPad Prism 7, Adobe Photoshop, and ImageJ Software. Treated groups were compared using a one-way or two-way ANOVA analysis with Bonferroni correction) to naïve mice. Immunophenotyping statistical analysis was determined with the Kruskal-Wallis test followed by Dunns’ test. Correlation coefficients (R) were calculated by using the Pearson’s rank matrix. Differences with a P value of <0.05 were considered significant: *P<0.05; **P<0.005; ***P<0.001,****P<0.0001.

### Data Availability Statement

All sequencing data that support the findings of this study have been deposited in the National Center for Biotechnology Information Gene Expression Omnibus (GEO) and are accessible through the GEO Series accession number GSE176203. All other relevant data are included in the article/**Supplementary Material**, further inquiries can be directed to the corresponding authors.

## Results

### Construction and Characterization of Sindbis Carrying the SARS-CoV-2-Spike

**Figure 1:**
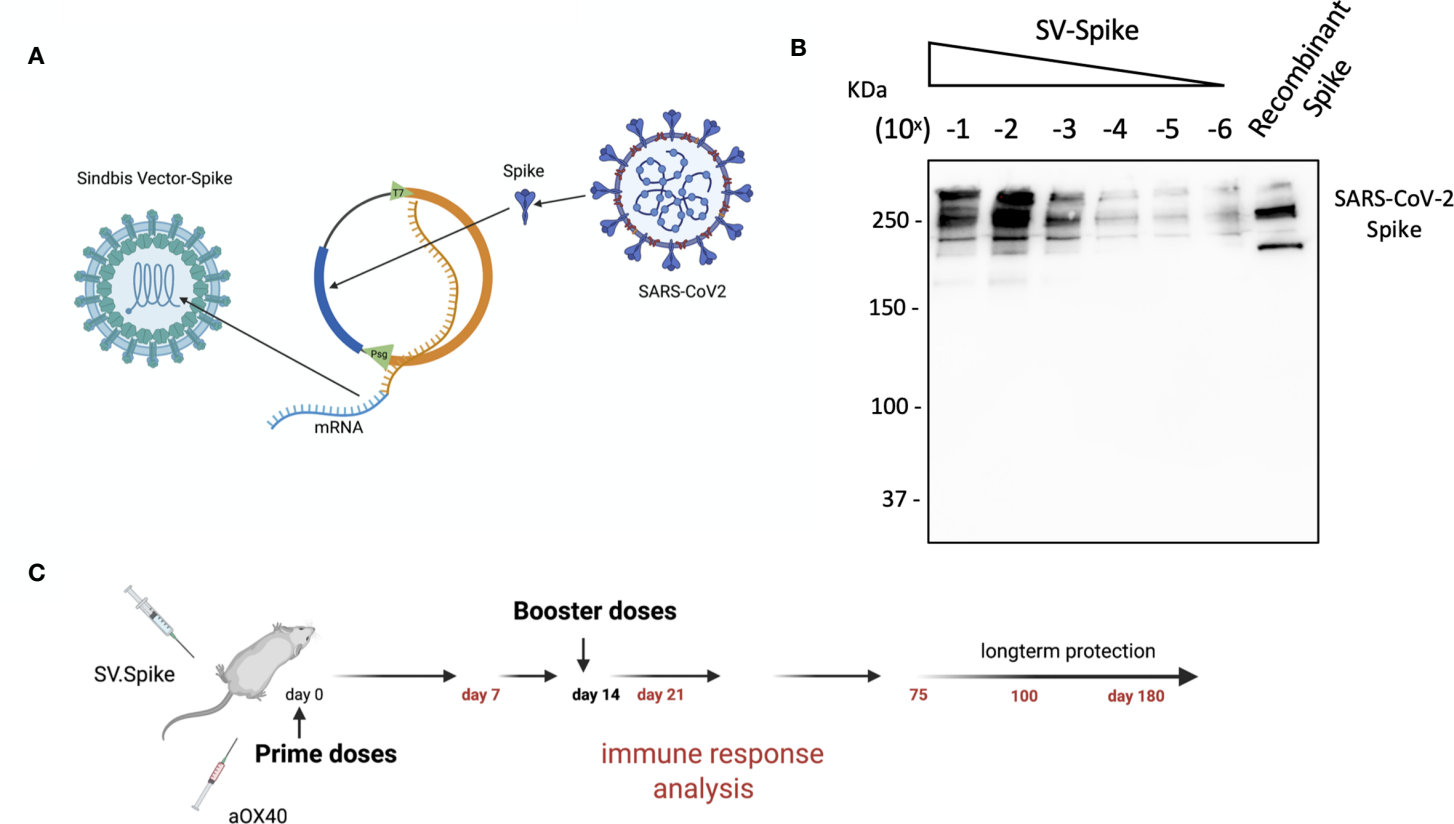
Characterization of Sindbis vector carrying the SARS-CoV-2 spike. **(A)** Schema of SARS-CoV-2 spike gene cloned into Sindbis vector system. **(B)** Western Blot of SARS-CoV-2 spike produced from the Sindbis vector. Lanes shown are titration of the vector, and recombinant spike control produced in HEK cells. **(C)** Schematic of vaccination. C57BL/6 mice were immunized with 1× 0.5 ml SV.Spike/and or αOX40 antibody (250 μg/dose) on day 0. A boost injection of SV.Spike/and or αOX40 were once given on day 14. On days 7, 14 and 21, 75, and 100, blood was taken to determine Sars-Cov-2 spike specific antibodies by ELISA. Spleens were excised and a single cell suspension was stained and analyzed by flow cytometry. T cells were isolated and were used for ELISPOT assay and Seahorse. As control, naïve C57BL/6J mice were used.

We designed and generated a Sindbis alphavirus replicon carrying the SARS-CoV-2 spike mRNA. SV vectors are generated from two plasmids: a replicon and helper (**Figure 1** and **Supplementary Figure 1**). Genes of interest (GOI) can be substituted for the 5kb structural genes that were removed to generate the helper plasmid. The plasmid encoding the structural genes does not contain a packaging signal, preventing further virus assembly beyond the initial preparation of the vectors in BHK-21 cells. Plasmids are transcribed from the T7 promoter and the RNA transcripts are electroporated into BHK-21 cells to produce viral vectors.

The combination of SV vectors encoding a selected antigen with immunomodulatory antibodies makes them far more effective than they are alone (35, 36, 56). In particular we have found that combining SV vectors expressing specific antigens with αOX40 generates very potent immune responses capable of eradicating tumors in multiple murine models and conferring long-term protection against tumor recurrences or rechallenges (36, 56).

The overall design in the production of Sindbis SARS-CoV-2 spike (SV.Spike) is illustrated in **Figure 1** and **Supplementary Figure 1**. We determined the expression of the full-length SARS-CoV-2 spike from infected cells by western blot in **Figure 1B**.

The immune responses induced by the Sindbis SARS-CoV-2 spike (SV.Spike) vaccine candidate were analyzed in C57BL/6J mice. Groups of mice (*n* = 5) were immunized by intraperitoneal (i.p.) or intramuscular (i.m) route, by prime-boost vaccine strategy with SV.Spike and/or αOX40, with 14 days difference between the two doses (**Figure 1C**). Immune response analysis from splenocytes, T cells, or mice sera was carried at different time points, as indicated in **Figure 1C**.

### Sindbis Vaccine-Elicited Antibodies to SARS-CoV-2 Spike

**Figure 2:**
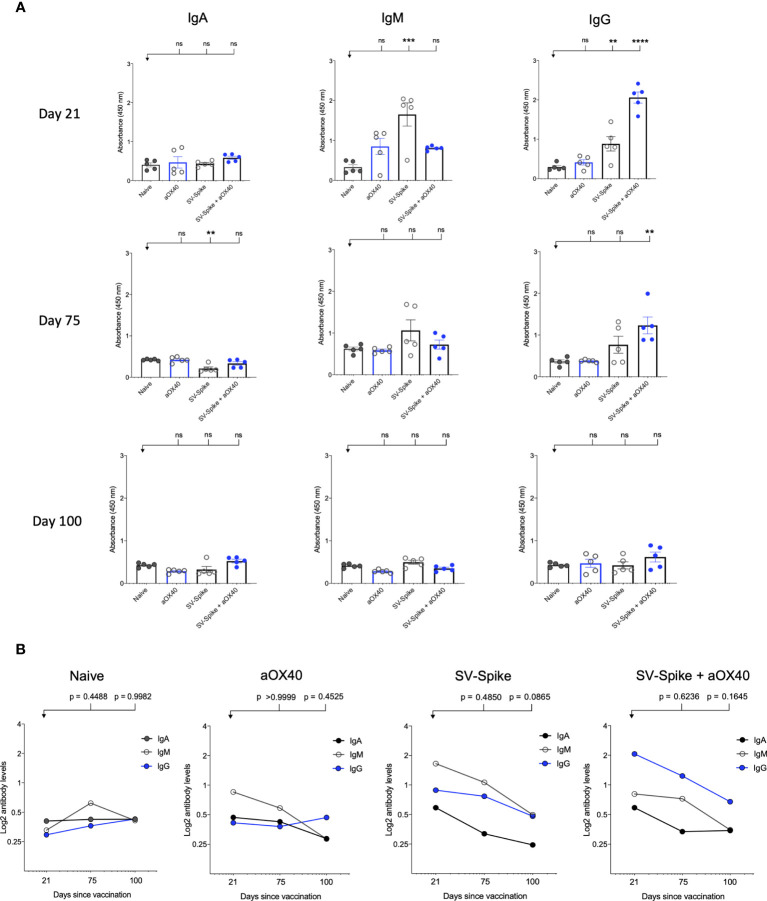
SARS-CoV-2 spike specific antibodies induced by Sindbis vector. Characterization of serum IgA, IgM, and IgG in C57BL/6J mice vaccinated with SV.Spike at days 21, 75, and 100 post-immunization. **(A)** The levels of Spike-specific IgA, IgM, and IgG isotypes in sera (diluted 1:100) of immunized mice at different time windows. P values were calculated by one-way ANOVA with the Bonferroni correction in Graphpad Prism. n.s. > 0.05; **P < 0.01; ***P < 0.001; ****P<0.0001. **(B)** The kinetics of Spike-specific IgA, IgM, and IgG isotypes in sera of immunized mice at different time windows. Mean comparison between time points and isotypes and relative P values were determined by two-way ANOVA with the Bonferroni correction in GraphPad Prism. The data presented are the mean of three technical replicates. The median values of **(A)** OD450 or **(B)** calculated log2 antibody levels were plotted for each isotype of three antibodies. Log2-scale in **(B)** was used to better represent the proportional relation between antibody titers and sera reciprocal dilution. The data presented are the mean of five biological replicates with two technical replicates.

Serum IgM, IgG, and IgA responses to SV.Spike, SV.Spike+αOX40, injections were measured on days 21, 75, and 100 days after vaccination by enzyme-linked immunosorbent assay (ELISA) against recombinant SARS CoV2 spike protein (11, 57). Sera from all of mice tested showed reactivity to recombinant SARS-CoV-2 spike protein and, as might be expected, levels of antibodies varied based on the experimental group and time point (**Figure 2**). Consistent with previous reports (58–60), levels of IgM and IgG measured at day 21 and 75 post injection (p.i.) were significantly higher in the mice vaccinated with SV.Spike and combination of SV.Spike+αOX40 than in the mice who had received αOX40 alone or the naïve group (**Figure 2A**). Moreover, the SV.Spike+αOX40 group showed higher titers of IgG compared with only SV.Spike treatment, for which IgM was the predominant isotype and did not show seroconversion to IgG over the different time points. Specifically, both SARS CoV2-specific IgG and IgM antibodies demonstrated the highest expression on day 21 post immunization for the indicated groups (IgG-OD450 of 2.3 for SV.Spike+αOX40 serum, and IgM-OD450 of 1.9 for SV.Spike serum). At days 75 p.i., IgG were still significantly predominant in the sera of the mice immunized with the SV.Spike+αOX40 combination (IgG-OD450 = 1.3), whereas IgM levels did not significantly vary from day 21 to day 100 compared with the control groups (**Figure 2B**). Instead, IgM levels in the SV.Spike mice showed a more significant decrease and less lasting reactivity from days 21 to 75 days p.i. (IgM-OD450 of 1.2) compared to the control group, whereas the IgG trend demonstrated significant high levels only at day 21 p.i. Conversely, IgA levels did not show any significant difference in any of the groups and time points tested (**Figures 2A, B** **)**. These data support the evidence that immunization of mice with SV.Spike combined with αOX40 elicits a strong and specific immune response compared to SV.Spike alone, which is predominantly represented by SARS-CoV-2 IgG- specific antibodies.

### Anti-SARS-CoV-2 Spike Neutralizing Antibodies Induced in Sindbis Vaccinated Mice Block the SARS-CoV-2 Spike Protein From Binding to hACE2 Receptor Proteins

Immediately after SARS-CoV-2 was identified as the causative agent of the COVID-19 outbreak, it was shown that human ACE2 (hACE2) is the main functional receptor for viral entry (61). We hypothesized that the virus–receptor binding can be mimicked *in vitro via* a protein–protein interaction using purified recombinant hACE2 and the SARS-CoV-2 spike protein. This interaction can be blocked by virus naturalizing antibodies (NAbs) present in the test serum of vaccinated mice.

**Figure 3:**
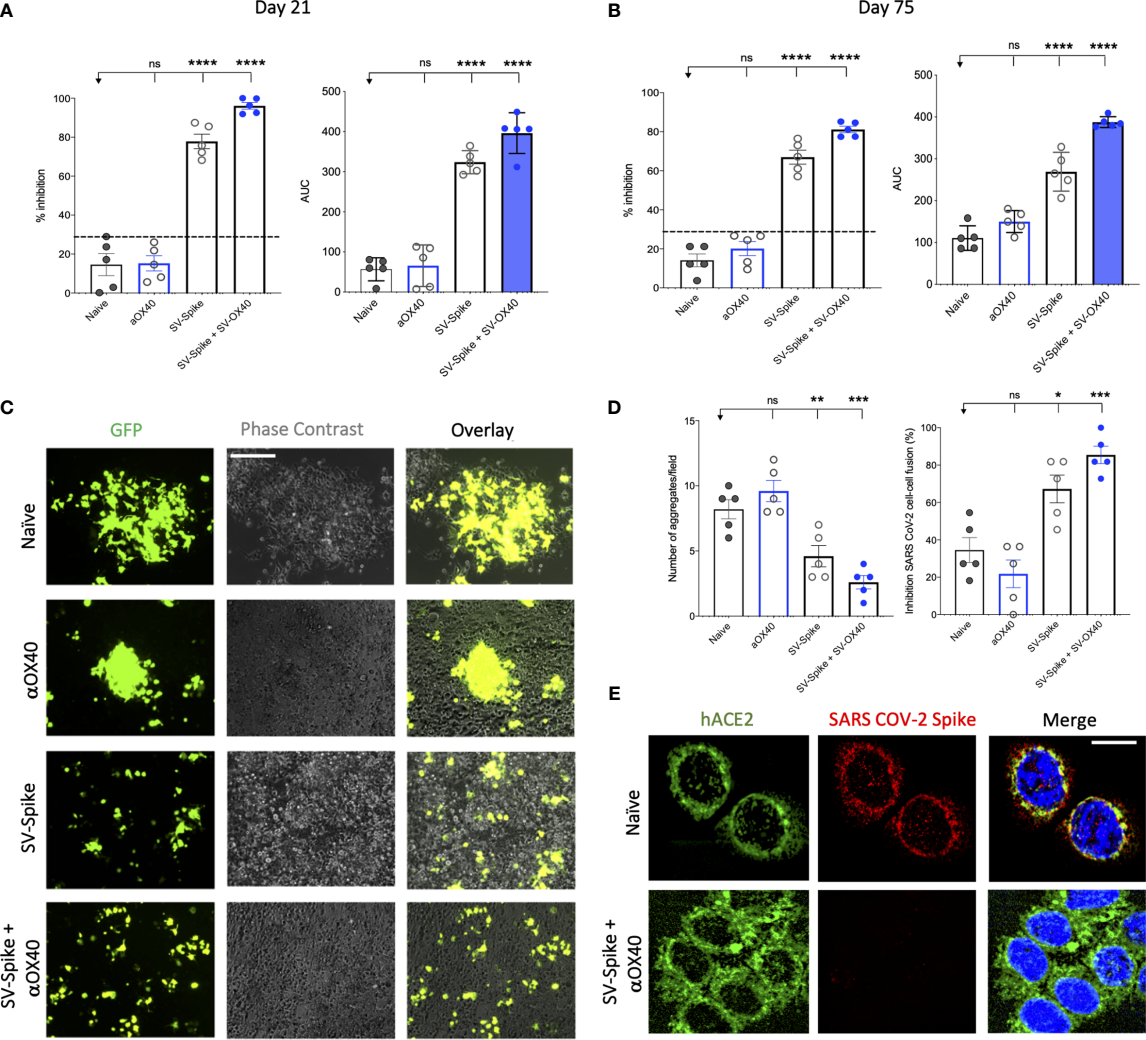
Blockade of SARS-CoV-2 spike-hACE2 binding and spike protein-mediated cell–cell fusion by anti-SARS-CoV-2 spike neutralizing antibodies. **(A, B)** In the assay, anti-SARS-CoV-2 neutralizing antibodies from immunized C57BL/6J mice, block recombinant Spike protein from binding to the hACE2 protein pre-coated on an ELISA plate. Percentage of inhibition distributed along y-axis of SARS-CoV-2 spike–hACE2 interaction for the indicated reciprocal plasma dilutions by mouse sera collected at **(A)** 21 and **(B)** 75 days post vaccination with Sindbis expressing SARS-CoV-2 spike (SV.Spike), SV.Spike in combination with αOX40 and αOX40 alone compared to the naive group. Area under the curve (AUC) values of serum antibodies were calculated from reciprocal dilution curves in antibody detection assay. The data presented are the mean of 5 biological replicates with two technical replicates. Statistics were performed using a One-way ANOVA with the Bonferroni correction in Graphpad Prism. n.s. > 0.05; *P < 0.05; **P < 0.01; ***P<0.001; ****P<0.0001. **(C)** Images of SARS-CoV-2 spike-mediated cell–cell fusion inhibition on 293T/ACE2 cells by sera from C57BL/6J vaccinated mice. SARS-CoV-2 spike-transfected 293T were incubated with mice serum at 1:100 dilution and applied onto 293T/ACE2 cells for 24 h. Scale bar: 100 µm. **(D)** Quantification of the number aggregates (left panel) and inhibition of cell–cell fusions (right panel) induced by SARS-CoV-2 spike following pre-incubation with naïve, SV.Spike, SV.Spike+αOX40 and αOX40 alone are shown. N = 5 biological replicates with two independent technical replicates. One-way ANOVA with Bonferroni correction *P < 0.05, **P < 0.01, and ***P < 0.001. **(E)** Representative confocal images of 293T/ACE2 cells treated with serum from Naïve and SV.Spike+αOX40-immunized mice pre-incubated with SARS-CoV-2 spike recombinant protein and stained for hACE2 (green), SARS-CoV-2 spike (red), and DAPI (blue). Scale bar: 20 μm.

A competition ELISA assay was developed to detect whether SARS-CoV-2 spike-specific antisera from mice immunized with αOX40, SV.Spike, and SV.Spike+αOX40 could block the interaction between SARS-CoV-2 spike and hACE2. Our assay demonstrated that the specific Spike–hACE2 binding can be neutralized by SV.Spike or SV.Spike+αOX40 sera in a dose-dependent manner, but not by sera from αOX40 alone or naïve groups (**Supplementary Figures 2A, B**). Similar results were obtained by the intramuscular route (**Supplementary Figure 2C**). As shown in **Figure 3A**, antibodies in the antisera from mice immunized with SV.Spike and combination of SV.Spike and αOX40 at day 21 post-immunization significantly inhibited the binding of SARS-CoV-2 spike to hACE2 compared to the sera from naïve mice, indicating that SV.Spike-induced antibodies could strongly neutralize SARS-CoV-2 infection by blocking the binding of Spike protein on the surface of SARS-CoV-2 to hACE2.

To investigate whether the neutralizing antibody response in immunized mice could maintain a high level for a longer period of time, we tested the neutralization activity of mice sera at 75 days post-immunization. The results showed that, although the overall antibody neutralizing capacity decreased compared to day 21, antibodies from SV.Spike and SV.Spike+αOX40 groups still significantly competed for the binding of the SARS-CoV-2 spike and hACE2 (**Figure 3B**), indicating that our SV.Spike vaccine is able to induce relative long-term neutralizing antibody responses.

Next, we investigated if the serum from mice immunized with SV.Spike could inhibit the cell membrane fusion process for viral entry (45, 46, 62) occurring upon the binding of SARS-CoV-2 spike Receptor Binding Domain (RBD) fragment to the ACE2 receptor on target cells. To establish an assay for measuring SARS-CoV-2-spike-mediated cell–cell fusion, we employed 293T cells (a highly transfectable derivative of human embryonic kidney 293 cells, that contain the SV40 T-antigen) expressing both SARS-CoV-2 spike and enhanced green fluorescent protein (EGFP) as effector cells and 293T cells stably expressing the human ACE2 receptor (293T/ACE2) as target cells. Notably, when the effector cells and the target cells were co-cultured at 37°C for 6 h and 24 h, the two types of cells started to fuse at 6 h, exhibiting a much larger size and multiple nuclei compared to the unfused cells. These changes were more significant at 24 h, resulting in hundreds of cells fused as one large syncytium with multiple nuclei that could be easily seen under both light and fluorescence microscopy (**Supplementary Figure 3**). The cell fusions were observed in the cells transfected with SARS-CoV-2 spike but not SARS-CoV Spike, whereas those cells transfected with EGFP only did not elicit such an effect, confirming that CoV-2 Spike-hACE2 engagement is essential for viral fusion and entry.

To determine whether the serum of mice immunized with SV.Spike can block Spike protein-mediated cell–cell fusion, we incubated the effector cells with serum from Naïve, SV.Spike, and/or αOX40 mice (diluted 1:100) at 37°C for 1 h and then we co-cultured them with the 293T/ACE2 target cells. We found that not only were fewer fusing cells observed, but also the size of fused cells were visually smaller in the groups of SARS-CoV-2-spike/293T effector cells pre-incubated SV.Spike with or without αOX40 sera compared to controls (**Figure 3C**). Quantification of fused cells per field in at least four randomly selected fields revealed a remarkably lower number of cell–cell fusions in both SV.Spike and SV.Spike+αOX40 groups compared to all the other groups. Moreover, SARS-CoV-2 spike-mediated cell–cell fusions were significantly inhibited by serum derived from SV.Spike+αOX40 vaccinated mice, indicating that addition of αOX40 to the vaccination protocol elicits antibodies with enhanced interference of syncytium formation mediated by SARS-CoV-2 infection (**Figures 3C, D** **)**.

The interference of immunized sera NABs with SARS-CoV-2-hACE2 binding was also determined by immunofluorescence experiments performed by culturing 293T/ACE2 cells with recombinant SARS-CoV-2 spike previously incubated with serum from naïve and SV.Spike and αOX40 immunized mice. The binding between Spike and hACE2 expressed on the cell surface was subsequently visualized *via* confocal fluorescence microscopy (**Figure 3E**). As expected, Spike incubated with SV.Spike+αOX40 serum was incapable of binding to hACE2, while the control group showed evident co-localization with hACE2 on the cell surface.

Taken together, these data demonstrate that SV.Spike sera alone and to a greater extent SV.Spike+αOX40 sera can neutralize SARS-CoV-2 spike-hACE2 interaction and in turn counteract virus entry mediated by cell-membrane fusion.

### SV.Spike Vaccine Prevents Infection of SARS-CoV-2 in Transgenic hACE2-Tg Mice

**Figure 4:**
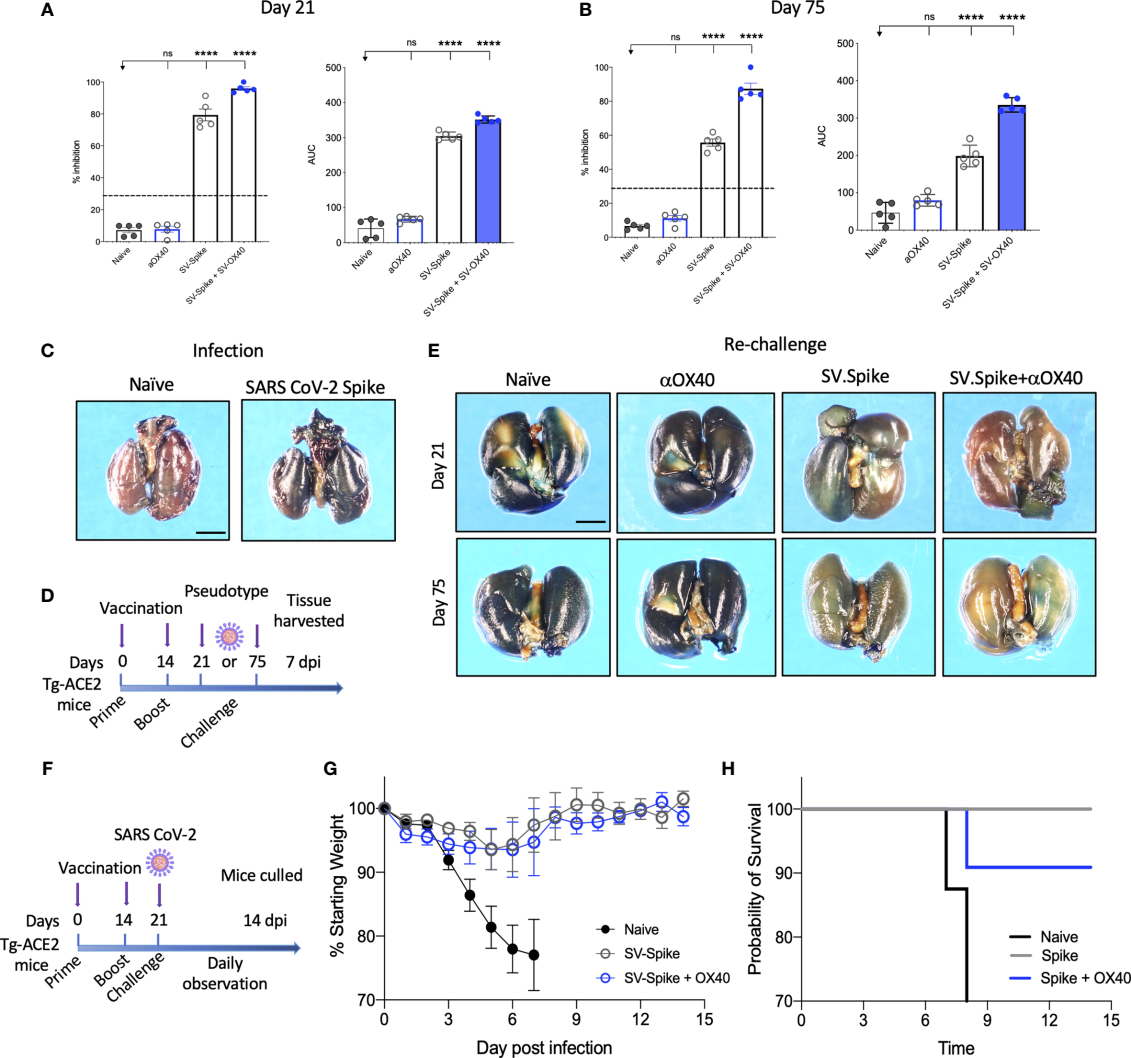
Sindbis-Spike vaccine prevents infection of SARS-CoV-2 in hACE2 transgenic (hACE2-Tg) mice. *Luciferase*-encoding SARS-CoV-2 spike pseudotyped lentivirus was incubated with mouse sera collected at **(A)** 21 and **(B)** 75 days post vaccination with SV.Spike, SV.Spike in combination with αOX40 and αOX40 antibody alone compared and unvaccinated naïve groups. Area under the curve (AUC) values of serum antibodies were calculated from reciprocal dilution curves in antibody detection assay. The data presented are the mean of 5 biological replicates with two technical replicates. Statistics were performed using a One-way ANOVA with the Bonferroni correction in GraphPad Prism. n.s. > 0.05; ****P<0.0001. **(C)** Expression of pseudotyped SARS-CoV-2-spike-lacZ lentivirus in whole mouse lung following intranasal delivery. One week following vector nasal administration to the right nostril of four weeks old hACE2 transgenic and control mice (B6(Cg)-Tg(K18-ACE2)2Prlmn/J), expression of LacZ was analyzed in mice airways. X-Gal stained whole lungs from (left) hACE2 non carrier control mouse and (right) hACE2 transgenic mouse, both dosed with SARS-CoV-2-spike-lacZ pseudotyped lentivirus. **(D)** Schematic of the “challenge” experiment with SARS-CoV-2-spike-lacZ lentivirus. **(E)** On day 21 (upper panels) and 75 (lower panels) after the initial infection hACE2-Tg were “challenged” with 3.6 x 10^5^ PFU of SARS-CoV-2-spike-lacZ pseudotyped lentivirus and then analyzed for X-Gal staining at day 7 “post-challenge.” Three non-vaccinated naïve animals were included as a positive control in the “challenge” experiment. **(F–H)** hACE2-Tg mice were vaccinated with SV.Spike and/or αOX40 and challenged with 10^4^ particles of live SARS-CoV-2 coronavirus at day 21 post immunization. Weight loss and mortality were observed daily for 14 days after live virus infection and compared to the naïve unvaccinated group. **(G)** Change of body weight during systemic infection with SARS-CoV-2 coronavirus. Percent weight loss (y-axis) is plotted versus time (x-axis). Data points represent mean weight change +/− SEM. **(H)** Survival curves of SV.Spike with or without αOX40 treated and naïve unvaccinated mice. n = 4 or 5 mice per group. One mouse belonging to the SV.Spike+aOX40 group died for inapparent reasons 7 days after challenge with the authentic SARS-CoV_2. Graph points in **(G)** were calculated as average of 4 mice instead of 5.

The neutralizing activity of serum from vaccinated mice was determined using Luciferase-encoding SARS-CoV-2 spike pseudotyped lentivirus (63–65) (**Supplementary Figures 5A, B**), by testing the impact of the serum on the lentivirus transduction. Serial dilutions (1:300, 1:600, 1:900: 1:1800, 1:3200, and 1:6400) of mice sera harvested at day 21 and 75 p.i. were incubated with equal amounts of lentivirus for 1 h at 37°C, then plated on 293T/ACE2 cells. We then measured the amount of blocked pseudotyped viral particles in infected cells by determining the amount of luminescence reduction, which reflects the level of neutralizing antibody or molecular inhibitors in the sample. The results showed that the antisera could inhibit SARS-CoV-2 pseudotype infection in a dose-dependent manner (**Supplementary Figure 5**), consistent with the result from the antibody neutralization assay (**Supplementary Figure 3**). Our results demonstrate that sera from SV.Spike with or without αOX40 immunized mice groups resulted in significantly high levels of neutralizing antibodies both at day 21 and 75, since they overcame the pseudotyped lentivirus infectivity inhibition threshold of 30% (**Figures 4A, B** **)**. Moreover, serum from these mice receiving combination of SV.Spike and αOX40 gave the highest levels of neutralization at day 21 after vaccination (95.3% of inhibition), with a slight decrease at day 75 (79% of inhibition). Naïve and αOX40 groups did not develop a neutralizing antibody response (% inhibition < 30%) at the timepoints tested, consistent with their lack of SARS-CoV-2 spike binding antibodies.

Recently, hACE2 transgenic (B6(Cg)-Tg(K18-ACE2)2Prlmn/J or hACE2-Tg) mice were used for the development of an animal model of SARS-CoV-2 infection (66). In order to test pseudotyped lentivirus infectivity rate *in vivo*, we produced a *nLacZ*-encoding lentivirus expressing SARS-CoV-2 spike protein (**Supplementary Figures 4B, D**) and we evaluated the vector expression following delivery to hACE-Tg mice airways, by administrating a single dose of *nLacZ-*pseudotype to 4-week-old hACE2-Tg mice by intranasal inhalation. After 7 days, the airways were harvested and intact glutaraldehyde-fixed tissues were processed for staining with X-Gal for detection of β-galactosidase activity expressed from the nuclear-localized lacZ reporter gene (*nlacZ;* **Figure 4C**). Positive X-Gal staining observed in airways upon lentivirus intranasal administration indicated the successful SARS-CoV-2-spike pseudotyped lentiviral vector expression and delivery in mice airways.

In order to investigate the protective effects of SV.Spike vaccination *in vivo*, we subsequently immunized hACE2-Tg mice with the same strategy as used for the C57BL/6J mice (**Figure 1D**). The hACE2-Tg mice were vaccinated at 0 and 2 weeks and then “challenged” with pseudotyped SARS-CoV-2 intranasally at day 21 and 75 post-immunization (**Figure 4D**). The lungs were collected at 7 days “post-challenge” and pseudotype delivery was tested by X-Gal staining. As shown in **Figure 4E**, the *nLacZ*-SARS-CoV-2-spike lentivirus could not be detected in the lungs from SV.Spike+αOX40 immunized mice, while substantially reduced infectious virus burden was still detected in the lungs from SV.Spike treated mice compared with the naïve group at the indicated time points. As expected, lungs from animals treated with αOX40 showed high amount of pseudotyped particles, as indicated from the very high signal of X-Gal staining (**Figure 4E**). Finally, protective immunity was also assessed in young adult vaccinated Tg-ACE2 mice challenged with live SARS-CoV-2 coronavirus. Three weeks after prime and boost vaccination doses, all mice were challenged with 10^4^ particles of SARS-CoV-2 *via* the intranasal (i.n.) route (**Figure 4F**). We recorded the daily the body weight of each mouse after infection for a total of 14 days and found that the body weights of both SV.Spike and SV.Spike+αOX40 mice showed a slow decrease at 3-5 days post infection (dpi), with a progressive stabilization, and increase of their weight at day 8-9 post infection. The naïve unvaccinated group showed a faster decrease during 3–5 dpi (**Figure 4G**), which led to early mortality around day 8 dpi (**Figure 4H**). Vaccinated mice did not evidence any signs of disease at the time the experiment was terminated but were culled on day 14 as required by the protocol, which was performed in an ABSL3 facility. Together, these data suggest that combination of SV.Spike and αOX40 vaccine in mice conferred long-term protection against SARS-CoV-2 infection by eliciting a durable humoral response in mice.

### SV.Spike in Combination With αOX40 Metabolically Reprograms and Activates T Cells Shortly After Prime Vaccine Doses

**Figure 5:**
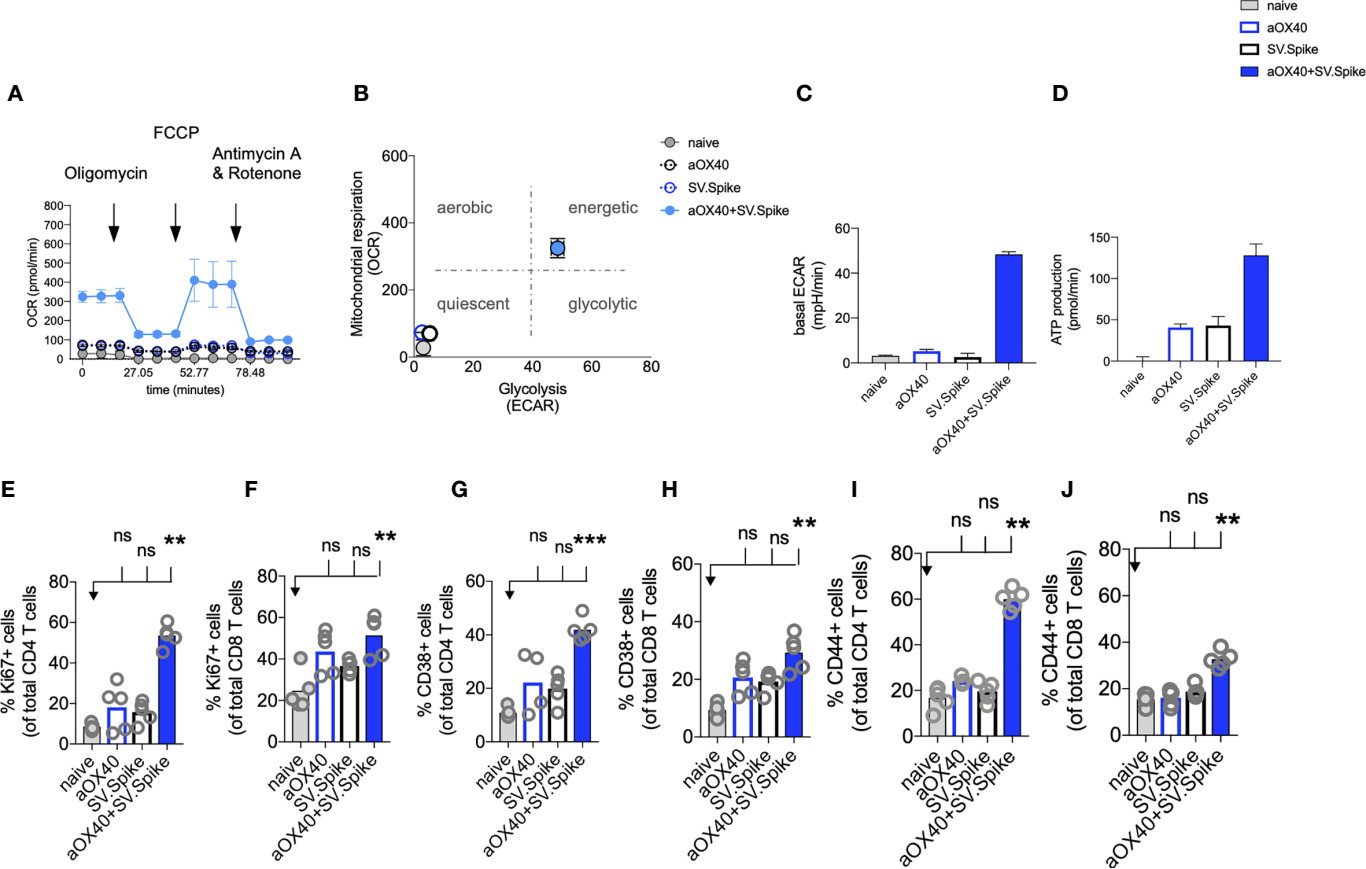
SV.Spike in combination with αOX40 activates and metabolically reprograms T cells. C57BL/6J mice were vaccinated with first doses of SV.Spike and/or αOX40. Naive mice were used as control. T cells were isolated from spleens on day 7 or otherwise indicated. **(A)** Mitochondrial respiration was assessed by measuring the median values of oxygen consumption rates (OCR) in T cells of indicated groups using an extracellular flux analyzer. Oligomycin, FCCP, Antimycin A, and Rotenone were injected as indicated to identify energetic mitochondrial phenotypes. **(B)** Energy map (OCR versus ECAR) of T cells from naïve or mice treated with SV.Spike, or αOX40 or combination of SV.Spike+αOX40 on day 7. **(C)** Baseline extracellular acidification rates (ECAR) in T cells of indicated groups. **(D)** ATP Production in T cells of indicated groups. **(E–J)** Splenocytes were analyzed in flow cytometry. **(E, F)** Expansion of CD4+ **(E)** and CD8+ T **(F)** cells is indicated by expression of Ki67-positive cells. **(G, H)** Activation of CD4+ T cells **(G)** and CD8+ T cells **(H)** indicated by CD38+ expression. **(I, J)** Expression of CD44+ positive cells. CD4 **(I)** and CD8 **(J)** cells. Error bars indicate SEM. Results are representatives of two independent experiments. Each symbol represents an individual mouse in **(E–J)** Bars represent means. Statistical significance was determined with the Kruskal-Wallis test followed by Dunns’ test. n.s. > 0.05, **p<0.005, ***p≤ 0.001.

Analysis of SARS-COV-2 specific adaptive immune responses during acute COVID-19 identified coordination between SARS-COV-2-specific CD4+ T cells and CD8+ T cells in limiting disease severity (67). We analyzed vaccine elicited T-cell responses in the spleen 7 days after mice received prime doses of SV.Spike and/or αOX40 and compared the initial T-cell response to naïve mice (**Figure 5**). Spleens of mice were excised and a single cell suspension was stained and analyzed by flow cytometry.

For a successful vaccine-elicited immune response, differentiation of virus-specific T cells from the naïve to the effector state requires a change in the metabolic pathways utilized for energy production (68). Therefore, metabolic profiles of vaccine-induced T cells are of interest and correlate to vaccine-mediated immunity (69).

We performed metabolic analysis of isolated T cells from spleens in an Extracellular Flux Analyzer XFe24 (Seahorse Bioscience) to investigate metabolic changes of T cells. We found, that combining our SV.Spike vaccine with agonistic αOX40 antibody metabolically rewires T cells *in vivo* shortly after initial vaccine doses (**Figures 5A–D**). T cells freshly isolated from mice on day 7 after first doses with SV.Spike+αOX40 combination displayed a metabolic shift to a highly bioenergetic state compared with single agent treatment or naïve mice that show a quiescent metabolism (**Figures 5A, B** **)**. Naïve T cells are quiescent and characterized by a metabolic program that favors energy production over biosynthesis. Upon T-cell receptor (TCR)-mediated stimulation, T cells become activated and metabolically reprogrammed. The bioenergetic state of metabolically reprogrammed T cells is characterized by a strong increase of oxygen consumption rate (OCR), which is a parameter for mitochondrial respiration (**Figure 5A**), and a strong increase of baseline extracellular acidification rate (ECAR) (**Figure 5C**), which is measured as a parameter for glycolysis. It has been shown that TCR signaling is directly tied to glycolysis (70). We found that T cells isolated from mice vaccinated with SV.Spike+αOX40 displayed a 3-fold increase of OCR and a 10-fold increase of ECAR compared to naïve and single agent vaccinated mice. T cells switched to the energetic state ramped up their ATP production (**Figure 5D**). A metabolic rapid adaptation is further required for effector T cells cytokine production and signaling. Rapid switch to type-1 cytokine production, such as IFN*γ* and granzyme B (GrB) in antiviral CD8+ T cells is more reliant on oxidative phosphorylation (71). Indeed, immunophenotyping of CD4+ and CD8+ T cells by flow cytometry revealed rapid clonal expansion of CD4+ T and CD8+ T subsets within one week after prime vaccine doses indicated by Ki67 expression on gated CD4+ and CD8+ T cells. As KI67 can be considered an indirect marker for T cell expansion, CD4+ T cells showed the highest expansion increase by 10-fold in the combination vaccinated group compared to naïve and SV.Spike and αOX40 single agent immunized mice (**Figures 5E, F** **)**. Both T-cell subsets were highly activated, indicated by CD38 and CD44 expression (**Figures 5G–J**) underlining successful vaccine elicited effector T-cell engagement by our vaccine shortly after initial vaccine doses. Similar results were obtained by the intramuscular route (**Supplementary Figure 6**).

### SV.Spike+αOX40 Vaccinated Mice Are Characterized by a Unique T-Cell Transcriptome Signature Profile After Prime Vaccine Doses

**Figure 6:**
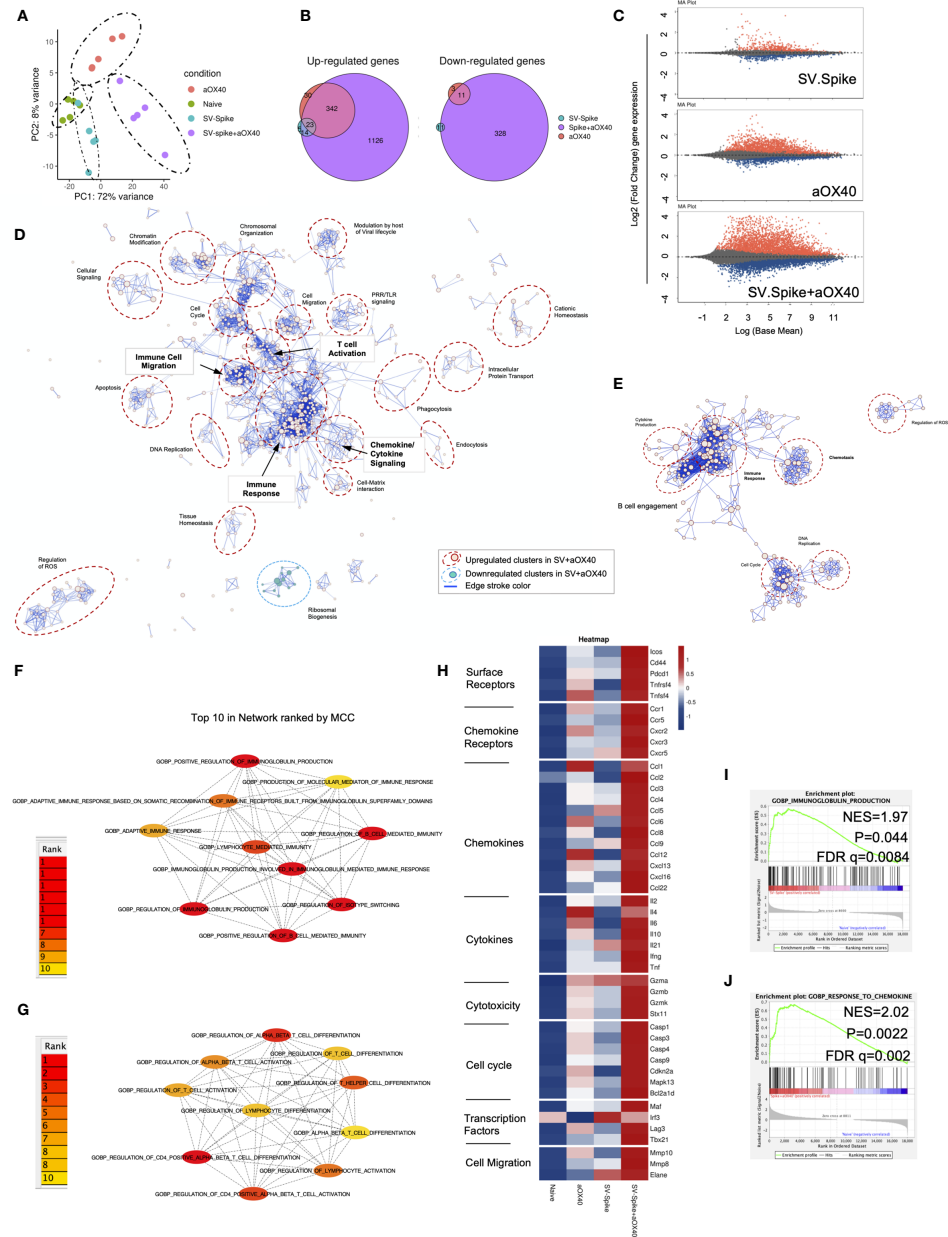
Sindbis expressing SARS-CoV-2 spike+αOX40 C57BL/6J vaccinated mice are characterized by a unique transcriptional signature of T cells. Combination therapy markedly changes the transcriptome signature of T cells favoring T-cell differentiation towards effector T-cells with a Th1 type phenotype 7 days after prime vaccination. **(A)** Principal component analysis (PCA) of RNA seq data from naïve, SV.Spike, and/or αOX40 groups. **(B)** Venn diagrams summarizing the overlap between differentially expressed genes (DEGs) from SV.Spike (blue), αOX40 (pink), and SV.Spike+αOX40 (purple). Up-regulated DEGs (left) and down-regulated (right). **(C)** MA plots of differentially expressed genes in T-cells of naive versus SV.Spike (top graph), αOX40 (middle graph), and combination (bottom graph). Significantly (p<0.05) upregulated and downregulated DEGs are depicted in red or blue, respectively. **(D)** Pathway and network analysis based on GSEA in T-cells isolated from mice treated with combination therapy. Downregulated (blue circle) and upregulated (red circles) pathways are shown, respectively. **(E)** Pathway and network analysis based on GSEA in T-cells isolated from mice treated with single dose of SV.Spike. Top 10 hub biological process gene ontology (GO) terms ranked by the Cytoscape plugin cytoHubba (red, highest ranks; yellow, lowest ranks) in the SV.Spike only **(F)** versus combination immunized group **(G)**. Heatmap analysis of selected genes based on normalized read counts linked to T-cell differentiation in the SV.Spike and/or αOX40 immunized mice compared to naïve **(H)**. Highlighted selected gene set enrichment analysis (GSEA) pathways based on DEG in naive versus SV.Spike **(I)** and combination treated group **(J)**.

To reveal the molecular profile of SV.Spike vaccine induced T-cell responses, we isolated T cells 7 days after prime vaccine doses from spleens of mice from SV.Spike and/or αOX40 vaccinated groups and naïve group. We then performed mRNA deep sequencing (RNAseq) and network analysis (**Figure 6**). Principal-component analysis (PCA) showed a distinct segregation between combined SV.Spike and αOX40 vaccination and all other groups (**Figure 6A**). These data suggest, that SV.Spike and αOX40 induces a distinct T-cell response. Indeed, we next looked at gene expression profiles of naïve versus SV.Spike and/or αOX40 and we found that naïve versus SV.Spike+αOX40 markedly showed the highest amount of uniquely upregulated and downregulated total genes with 1,126 upregulated (left) and 328 downregulated transcripts (**Figure 6B**). Overall, in all groups more genes were significantly upregulated than downregulated (**Figures 6B, C** **)**. These data suggest that SV.Spike+αOX40 changes the transcriptome signature of T cells. We performed Gene Ontology (GO) functional enrichment analysis (also Gene Set Enrichment Analysis, GSEA) and network analysis from naïve mice versus SV.Spike+αOX40 (**Figure 6D**) and naïve versus SV.Spike only (**Figure 6E**) immunized mice to determine key pathways and intersections of these pathways. The majority of pathways were upregulated in T cells isolated from mice immunized with SV.Spike+αOX40 with the exception of one cluster downregulated (ribosomal biogenesis). The upregulated pathways in the combination immunized mice were dominated by immune response, T-cell activation, chemokine/cytokine signaling, immune cell migration, DNA replication, chromosomal organization, cell cycle regulation, and chromatin modification that formed the central nodes of this network (**Figure 6D**). SV.Spike single agent immunized mice showed a smaller network of seven upregulated pathways including a main cluster of immune response closely connected to a cluster for to B-cell engagement, a small cluster of cytokine production, chemotaxis, cell cycle, DNA replication, regulation of ROS (**Figure 6E**).

We next identified the top 10 hub GO terms by employing the Maximal Clique Centrality (MCC) for SV.Spike (**Figure 6F**) and SV.Spike+αOX40 (**Figure 6G**) immunized mice. We found that top 10 hub GO terms in SV.Spike only immunized mice were a selected network cluster of B-cell stimulation and Immunoglobulin regulating pathways compared to the combination that represents a cluster of lymphocyte activation and differentiation regulating pathways. Additionally, we performed Protein Association Network Analysis using STRING to identify differentially expressed genes (DEGs)-encoded protein-protein interactions (PPIs). Significantly upregulated DEGs (log2FC>2, p<0.05) in T cells of SV.Spike and/or αOX40 vaccinated mice compared to naïve were analyzed to assess overrepresentation of Gene Ontology (GO) categories in Biological Processes in all groups (**Supplementary Figure 7**). GO Biological Processes (Strength ≥1; p<0.05) identified by STRING for each group were assigned to one of 7 clusters (apoptosis, light green; cell cycle, red; cellular signaling, dark blue; chemokines/chemotaxis, yellow; cytokines, pink; immune response, light blue; mitochondrial ATP production, dark green). Each GO Biological Process term is defined by one gene set. The amount of contributing DEGs from mice immunized with SV.Spike and/or αOX40 in each gene set is shown as percentage. We identified fourteen biological processes for αOX40, thirteen for SV.Spike, and forty-five for the combination vaccine strategy. We found cell cycle–related processes solely in the SV.Spike+αOX40 combination. The highest amount of chemokines/chemotaxis–related processes was observed in the combination (eleven) compared to αOX40 (four) and SV.Spike (four) alone. Six cytokine-related pathways were upregulated in the combination versus SV.Spike (one) and αOX40 (two) and 14 immune response-related terms were upregulated in the combination versus SV.Spike (four) and αOX40 (three). Overall, the percentage of DEGs that contribute to each biological process was highest in the combination vaccinated group compared to SV.Spike and αOX40 alone. Top 20 ranking of selectively enriched GO terms in the GSEA (FDA<0.05) revealed (GO) immunoglobulin production in the SV.Spike group (**Figure 6H**) and (GO) response to chemokine in the combination immunized mice group (**Figures 6I, J** **)**. We analyzed expression of single signature gene transcripts for each immunized mouse group. We found the highest upregulation of DEGs (p<0.05) indicating T-cell–dependent B-cell stimulation for building up humoral immunity against SARS-CoV-2 (*ICos, Cxcr5, Il21, Cxcl13*), differentiation of Th-1 type effector T cells associated with vaccine effectiveness (*Tnfrsf4, Cd44, ICos, Cxcr3, Ccr5, Il2, Ifng, Tbx21, Ccl3, Ccl4, Ccl9*) and antiviral cytotoxic T-cell stimulation for T-cell immunity (*Gzma, Gzmb, Gzmk*) in the SV.Spike+αOX40 immunized mice compared to single agent treated groups (**Figure 6H**).

In conclusion, these findings indicate that synergistic SV.Spike+αOX40 vaccine combination successfully changes the transcriptome profile of T cells that is indispensable for building up humoral and T-cell immunity.

### CD4+ T-Cell Help Promotes Effector Differentiation of Cytotoxic T Cells

**Figure 7:**
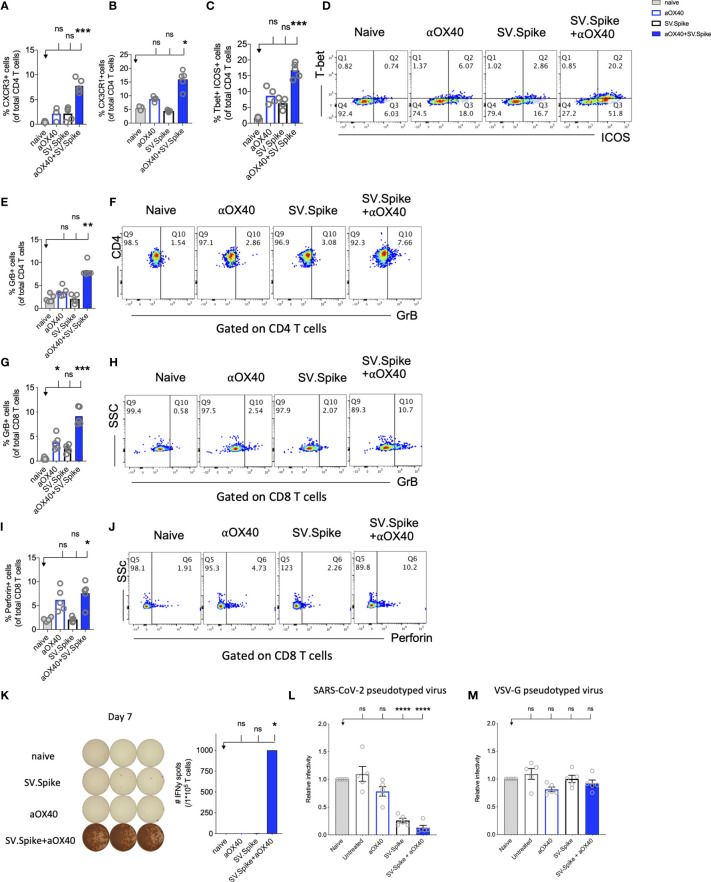
Reprogrammed T cells in SV.Spike+αOX40 vaccinated mice display enhanced Th-1 T-cell phenotype mediated cytokine production and cytotoxic T-cell activity. Spleens of C57BL/6J naïve and vaccinated mice were excised on day 7 after prime vaccine doses for flow cytometry analysis **(A–J)**. T cells were further isolated for **(K)** Interferon-g (IFN*γ*) enzyme-linked immunospot analysis (ELISpot) and **(L, M)** cytotoxicity analysis. Percentage of **(A)** CXCR3 and **(B)** CX3CR1 expressing CD4+ T cells indicating Th1-like T-cell effector phenotype. **(C)** Percentage of Tbet+ICOS+ positive Tfh-like effector CD4+ T-cell polarization. **(D)** Representative blots. **(E)** Percentage of granzyme B (GrB) positive CD4+ T cells from indicated groups using flow cytometry. **(F)** Representative blots. **(G)** Percentage of GrB positive CD8+ T cells from indicated groups using flow cytometry. **(H)** Representative blots. **(I)** Percentage of Perforin positive CD8+ T cells. **(J)** Representative blots. Bars represent means ± SEM (A–J) and each symbol represent an individual mouse (n=5 per group). Statistical significance was determined with the Kruskal-Wallis test followed by Dunns’ test. Results are representatives of at least two independent experiments. **(K)** Amount of IFN*γ* spots per 10^5^ T cells determined by ELISpot. **(L, M)** Cytotoxic activity of T cells harvested on day 7 from control and treated mice (n = 5 mice per group). T-cells were isolated from splenocytes and were co-cultured with 293T/ACE2 cells for 2 days. Effector-to-target (E/T) cell ratio (T cells/ACE2 cells) was 30:1. Cytotoxicity was determined for each group of mice by measuring the infectivity of luciferase-encoding pseudotyped particles with **(L)** Spike protein of SARS-CoV-2 or **(M)** VSV-G and is shown relative to naive T cells. Bars or symbols represent means ± SEM, and statistical significance was determined with one-way ANOVA with the Bonferroni correction. n.s. > 0.05, *p<0.05, **p<0.01, ***p≤ 0.001, ****p ≤ 0.0001.

CD4+ T helper cells are critical for success of vaccines and generally work by providing cytokines. Chemokine receptors help with the recruitment of type 1 effector and cytotoxic T cells to tissues and lymphoid organs, site- specific activation of memory T cells, and T-cell clustering around activated antigen presenting cells (APCs). For example, virus-specific cytotoxic T lymphocytes (CTLs) are quickly recruited to influenza-infected lungs by a Th1 response, specifically due to the production of IFN*γ* (72) Two of these Th1-type effector T-cell chemokine receptors are CXCR3 and CX3CR1. We performed flow cytometry analysis to investigate CD4+ T helper differentiation, formation, and antiviral cytotoxic effector T-cell differentiation in T cells from SV.Spike and/or αOX40 immunized animals (**Figure 7**). Vaccines mimicking an infection can help to build up immunity to the site of inflammation. Two of these Tfh-like effector T-cell chemokine receptors are CXCR3 and CX3CR1. We found a significant increase of CXCR3 and CX3CR1 positive expressing CD4+ T cells (**Figures 7A, B** **)** from spleens 7 days after administration of prime vaccine doses in the SV.Spike+αOX40 immunized mice group indicating mobility of generated Tfh-like effector T cells. Immunophenotyping by flow cytometry revealed a 2-fold increase of the transcription factor Tbet and immune costimulatory molecule ICOS-double-positive Tfh-like effector CD4+ T cells compared with single agent vaccinated mice. Tbet+ ICOS+ are hallmarks of Tfh-like T-cell polarization (**Figures 7C, D** **)**.

The predominant pathway used by human and murine CD8+ T cells to kill virus-infected cells is granule exocytosis, involving the release of perforin and GrB. It is known from influenza vaccine research that GrB correlates with protection and enhanced CTL response to influenza vaccination in older adults (73). We looked at CTLs after day 7 of prime doses and found that combination immunization significantly increased differentiation of CTLs as indicated by GrB+ expression (**Figures 7E–H**) and perforin (**Figures 7I, J** **)** upregulation within one week after initial vaccine doses. Seven days after mice groups received booster doses that were administered on day 14, we found a robust 10-fold upregulation of GrB+ positive CD8+ T cells indicating successful vaccine elicited differentiation of cytotoxic T cells (**Supplementary Figure 8**).

Interestingly, it has been reported that cytotoxic CD4+ T cells can compensate for age-related decline of immune cell protection such as B-cell loss and a less robust antibody response (74). Strikingly, we found in SV.Spike+αOX40 immunized mice showed a significant increase of cytotoxic CD4+ T cells indicating that our vaccine not only induced Tfh-like CD4+ T helper functions but has the potential to improve direct CD4+ T cell–mediated virus-killing, thus, adding an extra layer to immune protection against SARS-CoV-2 in more vulnerable older populations (**Figures 7E–J**). One important early feature of response to the SV.Spike+αOX40 immunization is a strong interferon-gamma (IFN*γ*) secretion (**Figure 7K**), which is associated with polarization to Tfh-like effector cells and cytotoxic T cells. To investigate the recruitment and specificity in CTLs to prevent SARS-CoV-2 cell entry, we analyzed the potential of T cells isolated from SV.Spike and/or αOX40 immunized and naïve mice on day 7 after prime doses to block the infection of 293T cells with SARS-CoV-2-spike expressing, luciferase-encoding pseudovirus. VSVG expressing, luciferase-encoding pseudovirus was used as control. We found that splenic T cells from SV.Spike and SV.Spike+αOX40 mice potently inhibited infection with SARS-CoV-2 pseudotyped lentivirus (**Figure 7L**) compared with VSV-G pseudotyped control T cells (**Figure 7M**). In conclusion, SV.Spike+αOX40 activated T cells display a Th-1 effector phenotype that promotes effector differentiation and direct T cell–mediated cytotoxicity against SARS-CoV-2 spike within one week after prime vaccine doses.

### SV.Spike in Combination With αOX40 Drives Metabolic Activation of B-Cells and T Cell–Dependent B-Cell Support

**Figure 8:**
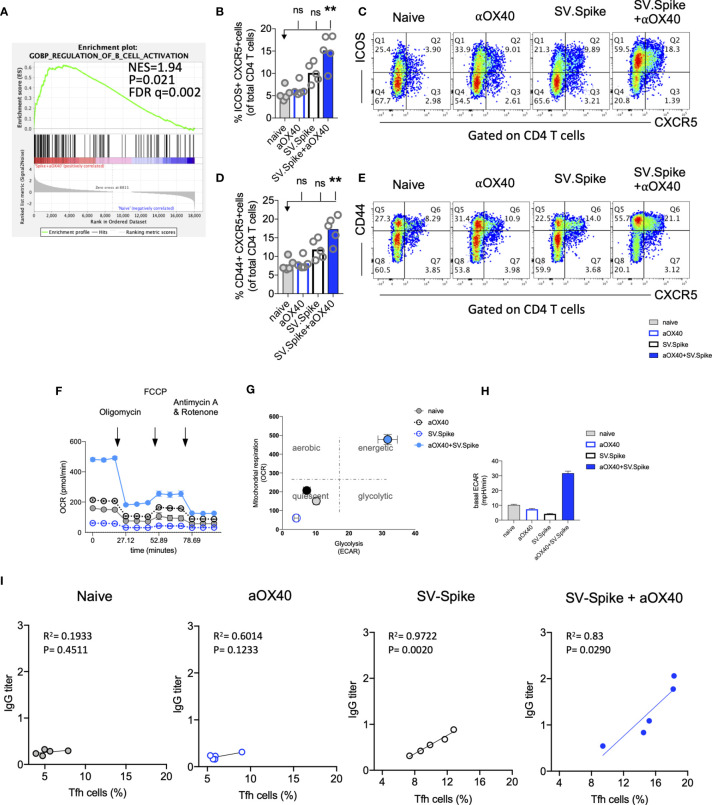
SV.Spike in combination with αOX40 drives follicular T helper cell function and metabolic activation of B-cells. C57BL/6J mice were vaccinated with SV.Spike and/or αOX40. Naive mice were used as control. T cells were isolated on day 7 after prime vaccine doses and RNAseq was performed **(A)**. GSEA for biological processes identified pathway enrichment that regulates B-cell activation after prime vaccine doses in combination immunized mice. Splenocytes were excised on day 21 for flow cytometry analysis **(B–E)**. **(B, C)** CXCR5+ICOS+ expressing CD4+ T cells and **(D, E)** CXCR5+CD44+ expressing CD4+ T cells indicating Tfh-cell differentiation with representative plots (n=5 individual mice per group). **(F–H)** B cells were isolated for Seahorse metabolic flux analysis one week after boost doses. **(F)** Mitochondrial respiration was assessed by measuring the median values of oxygen consumption rates (OCR) in B cells of indicated groups using an extracellular flux analyzer. Oligomycin, FCCP, Antimycin A, and Rotenone were injected as indicated to identify energetic mitochondrial phenotypes. **(G)** Energy Map (OCR versus ECAR) of B cells from naïve or mice treated with SV.Spike and/or αOX40 on day 21. **(H)** Baseline extracellular acidification rates (ECAR) in B cells of indicated groups. Error bars indicate SEM. Results are representatives of one or two independent experiments. Bars or symbols represent means ± SEM, and statistical significance was determined with the Kruskal-Wallis test followed by Dunns’ test. n.s. > 0.05, **p<0.005. **(I)** Correlation analysis of ICOS+CXCR5+ expressing Tfh cells with IgG antibody titers at 21 days post vaccination. (n=5). Pearson’s rank correlation coefficients (R) and p values are shown.

Almost all durable neutralizing antibody responses as well as affinity matured B-cell memory depend on CD4+ T-cell helper. GSEA of RNAseq data between T cells from the SV.Spike+αOX40 vaccinated and naive group one week after prime vaccine doses revealed selective enrichment of the gene set characteristic for activation of B-cells (**Figure 8A**) (p<0.05). To test if SV.Spike combination with αOX40 selectively regulates T cell–dependent B-cell activation, we investigated CD4+ T cell activation and differentiation in mice vaccinated with SV.Spike and/or αOX40 one week after booster vaccine doses by flow cytometry analysis. We found that SV.Spike+αOX40 immunized mice had a 3-fold significant increase of overall CD44+-positive splenic CD4+ T cells compared to naïve mice (**Supplementary Figure 9**). We next analyzed follicular CD4+ T helper (Tfh) cells that are a subset of CD4+ T cells required for most IgG responses promoting high-quality neutralizing antibodies and we found a 3-fold increase of ICOS+CXCR5+ (**Figures 8B, C** **)** and a 2 fold increase CD44+CXCR5+ (**Figures 8D, E** **)** positive CD4+ T cells in splenocytes from the SV.Spike+αOX40 group indicating Tfh cell differentiation. We isolated B-cells from spleens and performed a metabolic flux analysis on day 21 after initial vaccine doses and we found that isolated B-cells from SV.Spike+αOX40 immunized mice were metabolically reprogrammed indicating successful vaccine elicited B-cell activation (**Figures 8F–H**). Activated B-cells in the combination immunized group experienced a 2.5-fold increase in mitochondrial respiration (**Figures 8F, G** **)** and glycolysis (**Figures 8G, H** **)** when compared to B-cells isolated from mice spleens that were vaccinated with a single agent or compared to naïve mice. Association analysis of the frequencies of Tfh cells with SARS-CoV-2 spike IgG antibody titers revealed that Tfh cells positively correlated with the SARS-CoV-2 spike IgG serum levels in the SV.Spike (R^2^ = 0.9722, P=0.002) and SV.Spike+αOX40 group (R^2^ = 0.83, P = 0.0290) with the highest amounts of IgG antibodies and Tfh cells in the combination (**Figure 8I**). Taken together, these results indicate SV.Spike+αOX40 vaccine induced the most potent T cell–dependent B-cell response.

### Combination of SV.Spike and αOX40 Promotes Robust T Cell Immune Response in Lungs

**Figure 9:**
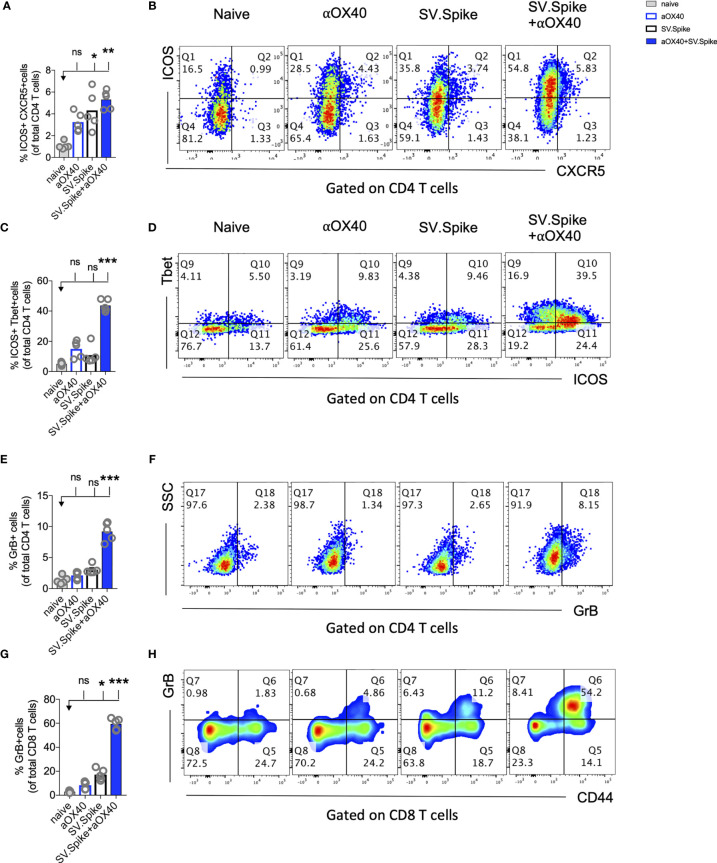
Combination of SV.Spike and αOX40 promotes robust tissue specific Tfh-like T cell immune response in lungs. Presence of activated T cells in lungs after 21 days after prime vaccine doses indicate tissue specific immune protection. C57BL/6J mice were immunized by a Prime/Boost strategy with SV.Spike and/or αOX40 and lungs were excised and a single cell-suspension was stained for flow cytometry analysis. Naive mice were used as control. **(A)** CD4+ Tfh type T cells presence in the lung indicated by ICOS+CXCR5+ double-positive CD4+ T cells. **(B)** Representative plots. **(C)** Expression of ICOS+Tbet+ double positive CD4+ T cells indicating Th-1 type effector cells polarization and recruitment to the lungs. **(D)** Representative plots. **(E–H)** Cytotoxic T cells in lungs indicated by **(E)** Granzyme B positive CD4+ T cells and representative plots **(F)** and CD8+ effector T cells indicated by GrB+ and representative plots **(G, H)**. Bars or symbols represent means ± SEM. Each symbol represents one individual mouse. Statistical significance was determined with the Kruskal-Wallis test followed by Dunns’ test. n.s. > 0.05, *p<0.05, **p<0.005, ***p≤ 0.001.

Most vaccines for airborne infectious diseases are designed for delivery *via* the muscle or skin for enhanced protection in the lung. We investigated if SV.Spike vaccine-induced T cells can readily home most efficiently to the lungs prior to and shortly after pathogen exposure. To address the immune responses in the lungs, we immunized mice with SV.Spike and/or αOX40 and excised PBS-perfused lungs one week after booster doses for single cell suspensions and performed flow cytometry staining (**Figure 9** and **Supplementary Figure 9**). We found an increase of ICOS+ CXCR5+ double-positive T helper cells indicating presence of B-cell supporting Tfh cells in the SV.Spike single agent and combination immunized group. We further found an increase of Th-1 type effector CD4+ T cells in lungs from combination treated mice indicated by expression of ICOS+Tbet+ double-positive effector CD4+ T cells (**Figures 9C, D** **)**. We next investigated if effector CTLs were successfully recruited into the lungs after 3 weeks of initial vaccine administration. While we found the highest increase of differentiated cytotoxic CD4+ T and CD8+ T cells in lungs from the combination treated group (**Figures 9E–H** and **Supplementary Figure 8**), we observed a significant increase of differentiated cytotoxic CD8+ T cells homing in the lungs of the SV.Spike single agent immunized group, although this increase was less pronounced compared to the combination group. These data indicate a successful recruitment of vaccine mediated antiviral Tfh-like effector T cells to the lungs.

### SV.Spike and αOX40 Promotes CD4+ T-Cell Memory Formation and Long-Term Protection Upon Recall With SARS-CoV-2 Spike Antigen

**Figure 10:**
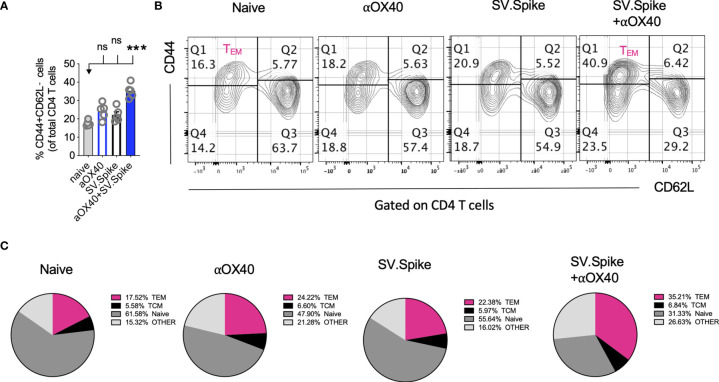
Combination of SV.Spike and αOX40 potentiates CD4 effector memory T cells 14 weeks after prime vaccine doses. Splenocytes from indicated immunized C57BL/6J mice groups were harvested 14 weeks after first vaccine doses. Memory phenotype was characterized in spleen from indicated groups by flow cytometry by gating on CD4+ cells. The percentage of effector CD4+ memory T cells, indicated by CD44+ CD62L−CD4+ markers, was analyzed and shown **(A)**. **(B)** Representative contour plots and **(C)** pie charts. (n=5 mice per group). TCM= CD44+CD62L+ double positive central memory T cells; TEM= CD44+ CD62L− effector memory T cells; naïve= CD44− but CD62L+ T cells, other= CD44− and CD62L− T cells. n.s. > 0.05, ***p≤0.001.

Boosting both, local and systemic memory T-cell response is a useful strategy to achieve long term immunity. We analyzed development of T-cell memory in spleens fourteen weeks after initial prime vaccine doses of SV.Spike and/or αOX40 prime-boost immunized mice by flow cytometry. We found that mice in the SV.Spike+αOX40 combination group developed significant effector CD4+ T memory indicated by CD44+ CD62L− CD4+ T cells (**Figures 10A–C**) compared with naïve mice, reiterating the importance of the combination vaccination in generating strong immune responses memory protection from infection and/or disease against SARS-CoV-2.

**Figure 11:**
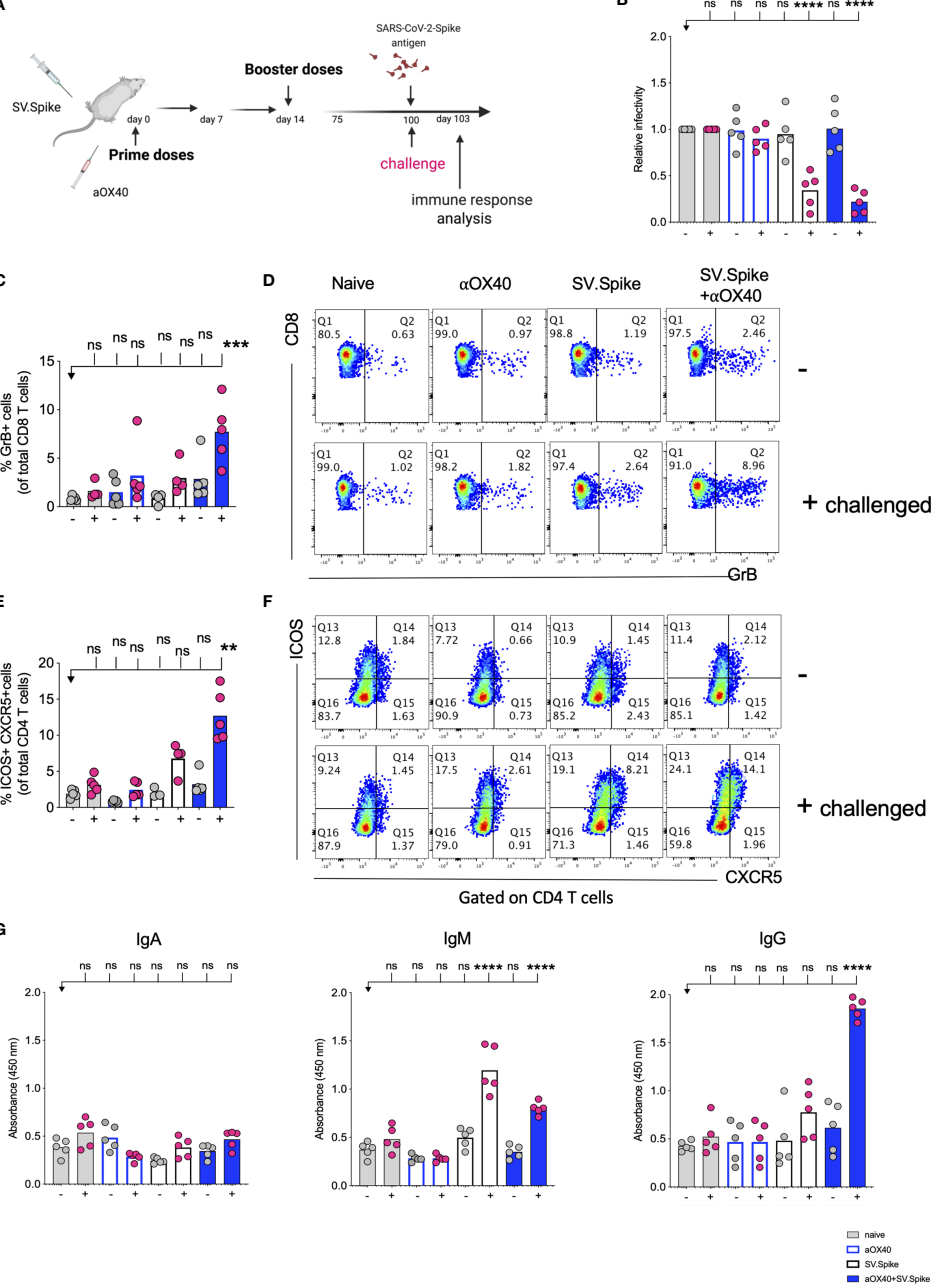
Challenging immunized mice with spike antigen promotes a fast and coordinated response of the two arms of the adaptive immune system. Humoral and T cell immune responses were assessed in vaccinated mice after “challenge” with Sindbis carrying SARS-CoV-2-spike (SV.Spike). **(A)** Design steps of the “challenge” experiment in immunized C57BL/6J mice evaluated by **(B)** T cell cytotoxic assay, **(C–F)** Flow cytometry indicating cytotoxic CD8 T cell effector response by GrB+ positive CD8 T cells and activation of CXCR5+ICOS+ positive Tfh cells upon “challenge”, **(G)** binding IgA, IgM, IgG antibody ELISA to SARS-CoV-2-spike recombinant protein (n=5 mice per group, or as otherwise indicated). Each symbol represents one individual mouse. Bars or symbols represent means ± SEM, and statistical significance was determined with one-way ANOVA with the Bonferroni correction **(B, G)** or with the Kruskal-Wallis test followed by Dunns’ test **(C–F)**. n.s. > 0.05, **p<0.005, ***p≤ 0.001, ****p ≤ 0.0001.

To further explore the long-term protection efficacy of our SV.Spike vaccine against SARS-CoV-2 virus exposure, C57BL/6J mice (n = 5 each group) received prime and boost immunizations of SV.Spike and/or αOX40 and placebo (naïve group) *via* the i.p. route. At day 100 post-immunization, we additionally administered one dose of SV.Spike, to recapitulate spike antigen endogenous entry through SV vector injection (**Figure 11A**). Spleens or sera from “challenged” mice were collected 3 days after SARS-CoV-2 spike antigen injection and processed for T-cell response analysis (**Figures 11B–F** and **Supplementary Figure 10**) and detection of specific anti-spike protein IgA, IgM, and IgG isotypes by ELISA (**Figure 11G**). The SARS-CoV-2 pseudotyped lentivirus infectivity assay revealed that mice immunized with SV.Spike or SV.Spike and αOX40 are effective in reactivating circulating cytotoxic T cells (CTLs) upon “challenge” with spike antigen (**Figure 11B**). CTLs reactivation was also observed by flow cytometry as indicated by granzyme B upregulation in mice receiving combination vaccination (**Figures 11C, D** **)**. Moreover, immunophenotyping analysis showed that CXCR5-ICOS-double-positive Tfh like effector CD4+ T cells were increased in “challenged” mice receiving SV.Spike combination vaccination compared to the same group of “unchallenged” mice (**Figures 11E, F** **)**.

Antibody response analysis showed that immunization with SV.Spike or SV.Spike+αOX40 followed by Spike antigen injection induced strong production of IgM antibodies compared with the mice that did not received the antigen and the naïve groups. This was particularly evident in mice vaccinated with SV.Spike (**Figure 11G**). Strikingly, we noticed that combination of SV.Spike and αOX40 followed by “challenge” with antigen stimulated a high peak of spike-specific IgG antibodies levels that were about 4 times higher than the IgG levels of “unchallenged” mice and control group mice. No significant difference in the spike-specific IgG response was detected in SV.Spike or single αOX40 “challenged” mice compared with the respective “unchallenged” mice and the control groups, whereas no SARS-CoV-2 spike-specific IgA was not detected in any of the groups (**Figure 11G**). To corroborate and validate our previous data, C57BL/6J mice (n = 5 each group) were “challenged” with SV.Spike at 180 days after receiving prime and boost immunization *via* the intramuscular (i.m.) route (**Supplementary Figure 11**). Immunophenotyping analysis of splenocytes isolated 3 days after recall antigen showed a remarkable increase in cytotoxic CD8 T-cell effector response in “challenged” mice which received vaccine combination compared to corresponding “unchallenged” mice, as indicated by granzyme B positive CD8 and CD4 T cells (**Supplementary Figure 11B**). Moreover, immunization with SV.Spike and aOX40 resulted in activation of effector CD4+ memory T cells, as indicated by CD44+ CD62L− CD4+ T cells upon “challenge” (**Supplementary Figure 11C**). Additionally, IgG seroconversion (**Supplementary Figure 11D**) and neutralizing antibodies (**Supplementary Figure 11E**) levels 180 days after immunization with vaccine combination were found significantly higher in mice exposed to spike antigen compared to the “unchallenged” and naïve control groups. Together, these data suggest that combination vaccination with SV.Spike and αOX40 conferred remarkably long-term and specific protection against SARS-CoV-2 infection by eliciting a durable humoral and T-cell response, regardless of the vaccine administration route.

## Discussion

An ideal vaccine against SARS-CoV-2 would be effective after one or two immunizations, conferring long-term protection to target populations such as the elderly or immunocompromised individuals, and reducing onward transmission of the virus to contacts (75). It would protect against a broad range of coronaviruses and evolving variants, i.e., offer pancoronavirus protection. The benefit of developing such a vaccine would be even greater if it were available to be rapidly deployed in time to prevent repeated or continuous epidemics, economical and readily distributable worldwide without temperature constraints that limit access. This supports the use of alphavirus vaccine platforms that are rapid and straightforward to produce inexpensively, with less challenging temperature requirements, and with previously proven safety and efficacy (76–79).

The alphavirus-based replicon platform technology has been developed as vaccine candidates for many different infectious diseases, including influenza A virus (IAV), respiratory syncytial virus (RSV) (80, 81) Ebola (EBOV), hepatitis C virus (HCV), chikungunya (CHIKV, now in phase III) (82, 83) HIV (now in phase I), human papilloma virus (HPV, now in therapeutic phase II) (84). Given the generic design of these platform and that new constructs can be made rapidly with synthetic design of the insert, it can be readily adapted to SARS-CoV-2 as we have demonstrated here. Moreover, when new virus species emerge, a vaccine platform that can be rapidly adapted to emerging viruses is highly desirable.

Sindbis and other alphaviruses have a natural tropism for lymphatic tissues and dendritic cells, relative resistance to interferon, high expression levels, lack of pre-existing anti-vector immunity in most human and animal populations, and efficient production in a broad range of cell lines, with an accepted regulatory pedigree (85). These observations indicate that a vaccine platform based on Sindbis virus vectors could contribute significantly to dealing with current and future vaccine needs. In nature SV has the safest profile among alphaviruses. SV is an RNA virus without replicative DNA intermediates and poses no risk of chromosomal integration or insertional mutagenesis. Hence, its presence is transitory. To avoid even transient adverse effects, our vectors have been attenuated by splitting the SV genome and by removing the packaging signal from the genomic strand that encodes the structural genes. Moreover, the combination of SV vectors with immunomodulatory antibodies like αOX40 makes them extremely effective compared to the single vector treatments (86, 87).

Neutralizing antibodies (NAbs) have conventionally been the desired outcome of vaccination, as they are capable of intercepting and neutralizing microbes and their components as well as eliciting destructive anti-microbial innate immune responses (88). Nonetheless, humoral immunity can decline over time and, as seen with influenza, can only last as short as one season. Many newer vaccines and vaccines in development are also designed to generate T-cell responses that have the potential to help the antibody response, promote long-term immune memory, have direct effector functions themselves, or activate innate effector cells such as macrophages and neutrophils (60, 89).

Here, we developed a Sindbis-based Spike-encoding RNA vaccine against SARS-CoV-2 and demonstrated that immunization with SV vector expressing SARS-CoV-2 spike along with a costimulatory agonistic αOX40 antibody induced a synergistic T cell and antibody response. Our adaptable approach has the potential to boost tissue specific immunity and immune memory against respiratory viruses and aims to develop vaccines with long-term protection. As a viral vector, we found that a Sindbis vector expressing SARS-CoV-2 spike antigen in combination with αOX40 markedly improves the initial T-cell priming, compared with the viral vector alone, which results in a robust CD4+ and CD8+ T-cell response and stable SARS-CoV-2 specific neutralizing antibodies, compared with the viral vector alone. The vaccine efficiently elicits effector T-cell memory in respiratory tissues with a potential for long lasting protection against COVID19 through multiple beneficial mechanisms. Importantly, it provided complete protection against authentic SARS-CoV-2 challenge in hACE2 transgenic mice preventing morbidity and mortality.

It has been shown that OX40 controls survival of primed CD8+ T cells and confers CTL-mediated protection (19, 90). CTLs are a critical component of the adaptive immune response but during aging, uncoordinated adaptive responses have been identified as potential risk factors that are linked to disease severity for the outcome of COVID19 patients. It is known from influenza vaccine research that granzyme B correlates with protection and enhanced CTL response to influenza vaccination in older adults. We looked at cytotoxic T cells (CTLs) and found that combination vaccination significantly increased CD8+ cytotoxic T cells indicated by granzyme B and perforin upregulation. A limitation of the present study is the lack of demonstration of antigen specific T cells. However, the nature of the T-cell response has been investigated through impact from the pseudotyped and authentic SARS-CoV-2 experiments. Almost all durable neutralizing antibody responses as well as affinity matured B-cell memory depend on CD4+ T helper cells. Our data indicates that agonistic OX40 antibody facilitates class switching from SARS-CoV-2 IgM and IgG antibodies in combination with SV.Spike. Moreover, in combination vaccinated mice, we found a significant increase of cytotoxic CD4+ T cells indicating that our vaccine not only induced CD4+ T helper functions but has the potential to improve direct CD4+ T cell−mediated virus-killing adding an extra layer to long-term immunity/protection in more vulnerable older populations.

Virus-specific CTL are quickly recruited to influenza-infected lungs by a Th1 response, specifically due to the production of IFN*γ* (72). IFN*γ* regulates various immune responses that are critical for vaccine-induced protection and has been well studied (91, 92). In a clinical trial of the now approved BNT162b1 COVID19 vaccine, IFN*γ* secreting T cells increased in participants 7 days after boost (60). In this regard, one important early feature of the response to the SV.Spike+αOX40 immunization is a strong interferon-gamma (IFN*γ*) secretion compared to naïve and single agent immunized mice. We found a significant increase of CXCR3 and CX3CR1 positive expressing CD4+ T cells, indicating effective recruitment and mobility of generated effector Th1 type T cells in mice. The presence of Th1-type effector T cells in the lungs indicates mobility of vaccine induced activated T cells/tissue surveillance, which positively correlates with vaccine induced long-term immune protection and generation of neutralizing antibodies against SARS-CoV-2.

Both humoral and cell-mediated immune responses have been associated with vaccine-induced protection against challenge or subsequent re-challenge after live SARS-CoV-2 infection in recent rhesus macaque studies (93, 94) and there is mounting evidence that T-cell responses play an important role in COVID-19 mitigation (11, 95, 96). We demonstrated that two doses of SV.Spike with or without αOX40 candidate vaccines induced neutralizing antibody titers in all immunized mice, with a strong IgG response in the mice receiving combination vaccination. Moreover, our data show that SV.Spike+αOX40 skewed Tfh cells toward CXCR5^+^ Tfh differentiation, which positively correlated with the magnitude of IgG isotype response. These findings indicate that the induction of CXCR5^+^ Tfh cell differentiation through vaccination may be beneficial for eliciting broad and specific NAb responses. Importantly, the synergistic activity of combination vaccination elicited antibodies that were able to efficiently neutralize SARS-CoV-2 pseudotyped lentivirus in all the mice tested. In addition, we show SV-Spike-based recall in mice immunized with combination vaccination led to an increase in cytotoxic T cells and IgG seroconversion and response 3 days only after exposure to recall antigen compared to all other and “unchallenged” groups, reiterating the importance of the involvement of both humoral and cellular immune responses in SARS-CoV-2-mediated immunity.

The SV.Spike platform evaluated in this study has the advantage that it is inexpensive, stable, and easy to produce. Cost projections based on using our upstream and downstream processes for production of a SV based vaccine are in line with or below costs per dose for other vaccines in use today. Moreover, unlike other mRNA vaccine candidates this viral platform does not require a cold-chain during transportation and storage. It can be easily reconstitute after lyophilization process and is suitable for rapid adaptation such that potential new viruses/threats in an emerging outbreak can be rapidly targeted (97). Thus, for emerging pathogens like SARS-CoV-2, the SV platform can be an efficient and cost-effective alternative to the traditional large-scale antigen production or technology platforms that require extended time for implementation. Development of a successful SV vector vaccine is readily translatable into human vaccination efforts.

As shown in this study, SV.Spike can be applied alone or can be combined with immunomodulatory reagents like αOX40 in a remarkably efficient prime-boost regimen.

Our goal is to exploit the combined SV.Spike+αOX40 formulation and integrate the two components into a single vector, to further facilitate administration and immunomodulatory response. Our lab has recently demonstrated that the expression of full-length antibodies from SV vectors is feasible and effective and that we can also integrate a third gene of interest such as an antigen or a cytokine (unpublished). Taken together, the overall data presented provide an insight into antigen design and preclinical evaluation of immunogenicity of SV-based vaccine immunogenicity, and support further development of SV.Spike as a vaccine candidate for protection against COVID-19 and pancoronavirus vaccine generation.

## Data Availability Statement

The RNAseq data has been uploaded to NCBI - accession number is GSE176203. Data and materials availability: Correspondence should be addressed to DM.

## Ethics Statement

The animal study was reviewed and approved by the Institutional Animal Care and Use Committee (IACUC) of New York University Grossman School of Medicine. Centers for Disease Control and Prevention (CDC), and the National Institutes of Health (NIH) for the authentic coronavirus experiments.

## Author Contributions

AS, SO, and DM conceived the study. AS, SO, AH, CP designed experiments. AS and SO performed mouse experiments and related data analysis. AH and CP designed the Sindbis vector. ZL processed and analyzed the RNAseq data. MN, ST, and KS performed BSL3 experiments with live coronavirus and related data analysis. AS, SO, and DM wrote the manuscript. All authors contributed to the article and approved the submitted version.

## Conflict of Interest

All authors are employed by NYU Langone School of Medicine and have no employment relationship or consultancy agreement with Cynvec a biotechnology company that support some studies under a Research and Licensing agreement with NYU. AS, SO, AH, ZL, CP, and DM are inventors on one or several issued patents and/or patent applications held by NYU that cover Sindbis treatment of neoplasia and COVID19. As part of the Research and Licensing agreement authors who are inventors on patents are entitled to a portion of NYU Langone’s royalties received, should Sindbis vectors be approved by the FDA for the therapeutic or vaccination use.

## Publisher’s Note

All claims expressed in this article are solely those of the authors and do not necessarily represent those of their affiliated organizations, or those of the publisher, the editors and the reviewers. Any product that may be evaluated in this article, or claim that may be made by its manufacturer, is not guaranteed or endorsed by the publisher.

## References

[1] Hoffmann M Arora P Groß R Seidel A Hörnich B Hahn A. SARS-CoV-2 Variants B.1.351 and B.1.1.248: Escape From Neutralizing Antibodies. Cell (2021) 184(9):2384–93.e12. 10.1016/j.cell.2021.03.03 PMC7980144 33794143

[2] Moore JP Offit PA. SARS-CoV-2 Vaccines and the Growing Threat of Viral Variants. JAMA (2021) 325:821–2. 10.1001/jama.2021.1114 33507218

[3] Wang P Nair MS Liu L Iketani S Luo Y Guo Y. Antibody Resistance of SARS-CoV-2 Variants B.1.351 and B.1.1.7. Nature (2021) 593:130–5. 10.1038/s41586-021-03398-2 33684923

[4] Collier DA De Marco A Ferreira IATM Meng B Datir RP Walls AC. Sensitivity of SARS-CoV-2 B.1.1.7 to mRNA Vaccine-Elicited Antibodies. Nature (2021) 593:136–41. 10.1101/2021.01.19.21249840 PMC7616976 33706364

[5] Wise J. Covid-19: The E484K Mutation and the Risks it Poses. BMJ (2021) 372:n359. 10.1136/bmj.n359 33547053

[6] Thompson RN Hill EM Gog JR. SARS-CoV-2 Incidence and Vaccine Escape. Lancet Infect Dis (2021) 21(7):913–4. 10.1016/S1473-3099(21)00202-4 PMC8043582 33861968

[7] D. The Lancet Infectious. An Exceptional Vaccination Policy in Exceptional Circumstances. Lancet Infect Dis (2021) 21:149–9. 10.1016/S1473-3099(21)00008-6 PMC7832878 33460564

[8] Available at: https://ig.ft.com/coronavirus-vaccine-tracker/?areas=gbr&areas=isr&areas=usa&areas=eue&cumulative=1&populationAdjusted=1.

[9] Wouters OJ Shadlen KC Salcher-Konrad M Pollard AJ Larson HJ Teerawattananon Y. Challenges in Ensuring Global Access to COVID-19 Vaccines: Production, Affordability, Allocation, and Deployment. Lancet (2021) 397:1023–34. 10.1016/S0140-6736(21)00306-8 PMC7906643 33587887

[10] Moore AC Hutchings CL. Combination Vaccines: Synergistic Simultaneous Induction of Antibody and T-Cell Immunity. Expert Rev Vaccines (2007) 6:111–21. 10.1586/14760584.6.1.111 17280483

[11] Grifoni A Weiskopf D Ramirez SI Mateus J Dan JM Moderbacher CR. Targets of T Cell Responses to SARS-CoV-2 Coronavirus in Humans With COVID-19 Disease and Unexposed Individuals. Cell (2020) 181:1489–501.e15. 10.1016/j.cell.2020.05.015 32473127 PMC7237901

[12] Paterson DJ Jefferies WA Green JR Brandon MR Corthesy P Puklavec M. Antigens of Activated Rat T Lymphocytes Including a Molecule of 50,000 Mr Detected Only on CD4 Positive T Blasts. Mol Immunol (1987) 24:1281–90. 10.1016/0161-5890(87)90122-2 2828930

[13] Calderhead DM Buhlmann JE van den Eertwegh AJ Claassen E Noelle RJ Fell HP. Cloning of Mouse Ox40: A T Cell Activation Marker That may Mediate T-B Cell Interactions. J Immunol (1993) 151:5261–71. 8228223

[14] Croft M. Control of Immunity by the TNFR-Related Molecule OX40 (Cd134). Annu Rev Immunol (2010) 28:57–78. 10.1146/annurev-immunol-030409-101243 20307208 PMC2882161

[15] Zander RA Obeng-Adjei N Guthmiller JJ Kulu DI Li J Ongoiba A. PD-1 Co-Inhibitory and OX40 Co-Stimulatory Crosstalk Regulates Helper T Cell Differentiation and Anti-Plasmodium Humoral Immunity. Cell Host Microbe (2015) 17:628–41. 10.1016/j.chom.2015.03.007 PMC4433434 25891357

[16] Wang Q Shi B-M Xie F Fu Z-y Chen Y-J An J-N. Enhancement of CD4+ T Cell Response and Survival via Coexpressed OX40/OX40L in Graves’ Disease. Mol Cell Endocrinol (2016) 430:115–24. 10.1016/j.mce.2016.04.008 27107937

[17] Zander RA Vijay R Pack AD Guthmiller JJ Graham AC Lindner SE. Th1-Like Plasmodium-Specific Memory CD4+ T Cells Support Humoral Immunity. Cell Rep (2017) 21:1839–52. 10.1016/j.celrep.2017.10.077 PMC5693336 29141217

[18] Gramaglia I Weinberg AD Lemon M Croft M. Ox-40 Ligand: A Potent Costimulatory Molecule for Sustaining Primary CD4 T Cell Responses. J Immunol (1998) 161:6510–7. 9862675

[19] Bansal-Pakala P Halteman BS Cheng MH Croft M. Costimulation of CD8 T Cell Responses by OX40. J Immunol (2004) 172:4821–5. 10.4049/jimmunol.172.8.4821 15067059

[20] Mousavi SF Soroosh P Takahashi T Yoshikai Y Shen H Lefrançois L. OX40 Costimulatory Signals Potentiate the Memory Commitment of Effector CD8+ T Cells. J Immunol (Baltimore Md: 1950) (2008) 181:5990–6001. 10.4049/jimmunol.181.9.5990 PMC2784683 18941188

[21] Tahiliani V Hutchinson TE Abboud G Croft M Salek-Ardakani S. OX40 Cooperates With ICOS To Amplify Follicular Th Cell Development and Germinal Center Reactions During Infection. J Immunol (2017) 198:218–28. 10.4049/jimmunol.1601356 PMC5173420 27895177

[22] Lindgren G Ols S Liang F Thompson EA Lin A Hellgren F. Induction of Robust B Cell Responses After Influenza mRNA Vaccination Is Accompanied by Circulating Hemagglutinin-Specific ICOS+ PD-1+ CXCR3+ T Follicular Helper Cells. Front Immunol (2017) 8:1539. 10.3389/fimmu.2017.01539 29181005 PMC5693886

[23] Cárdeno A Magnusson MK Quiding-Järbrink M Lundgren A. Activated T Follicular Helper-Like Cells Are Released Into Blood After Oral Vaccination and Correlate With Vaccine Specific Mucosal B-Cell Memory. Sci Rep (2018) 8:2729–9. 10.1038/s41598-018-20740-3 PMC5807513 29426881

[24] Linterman MA Hill DL. Can Follicular Helper T Cells be Targeted to Improve Vaccine Efficacy? F1000Res (2016) 5:F1000 Faculty Rev–88. 10.12688/f1000research.7388.1 PMC4784016 26989476

[25] Erdman MM Kamrud KI Harris DL Smith J. Alphavirus Replicon Particle Vaccines Developed for Use in Humans Induce High Levels of Antibodies to Influenza Virus Hemagglutinin in Swine: Proof of Concept. Vaccine (2010) 28:594–6. 10.1016/j.vaccine.2009.10.015 19853679

[26] Hevey M Negley D Pushko P Smith J Schmaljohn A. Marburg Virus Vaccines Based Upon Alphavirus Replicons Protect Guinea Pigs and Nonhuman Primates. Virology (1998) 251:28–37. 10.1006/viro.1998.9367 9813200

[27] Hooper JW Ferro AM Golden JW Silvera P Dudek J Alterson K. Molecular Smallpox Vaccine Delivered by Alphavirus Replicons Elicits Protective Immunity in Mice and non-Human Primates. Vaccine (2009) 28:494–511. 10.1016/j.vaccine.2009.09.133 19833247 PMC2789203

[28] Bernstein DI Reap EA Katen K Watson A Smith K Norberg P. Randomized, Double-Blind, Phase 1 Trial of an Alphavirus Replicon Vaccine for Cytomegalovirus in CMV Seronegative Adult Volunteers. Vaccine (2009) 28:484–93. 10.1016/j.vaccine.2009.09.135 19857446

[29] Diaz-San Segundo F Dias CCA Moraes MP Weiss M Perez-Martin E Owens G. Venezuelan Equine Encephalitis Replicon Particles can Induce Rapid Protection Against Foot-and-Mouth Disease Virus. J Virol (2013) 87:5447–60. 10.1128/JVI.03462-12 PMC3648198 23468490

[30] Reed DS Glass PJ Bakken RR Barth JF Lind CM da Silva L. Combined Alphavirus Replicon Particle Vaccine Induces Durable and Cross-Protective Immune Responses Against Equine Encephalitis Viruses. J Virol (2014) 88:12077–86. 10.1128/JVI.01406-14 PMC4178741 25122801

[31] Deming D Sheahan T Heise M Yount B Davis N Sims A. Vaccine Efficacy in Senescent Mice Challenged With Recombinant SARS-CoV Bearing Epidemic and Zoonotic Spike Variants. PloS Med (2006) 3:e525. 10.1371/journal.pmed.0030525 17194199 PMC1716185

[32] Pushko P Bray M Ludwig GV Parker M Schmaljohn A Sanchez A. Recombinant RNA Replicons Derived From Attenuated Venezuelan Equine Encephalitis Virus Protect Guinea Pigs and Mice From Ebola Hemorrhagic Fever Virus. Vaccine (2000) 19:142–53. 10.1016/S0264-410X(00)00113-4 10924796

[33] Lundstrom K. Alphavirus-Based Vaccines. Viruses (2014) 6:2392–415. 10.3390/v6062392 PMC4074933 24937089

[34] Granot T Yamanashi Y Meruelo D. Sindbis Viral Vectors Transiently Deliver Tumor-Associated Antigens to Lymph Nodes and Elicit Diversified Antitumor CD8+ T-Cell Immunity. Mol Ther: J Am Soc Gene Ther (2014) 22:112–22. 10.1038/mt.2013.215 PMC3978799 24025748

[35] Scherwitzl I Hurtado A Pierce CM Vogt S Pampeno C Meruelo D. Systemically Administered Sindbis Virus in Combination With Immune Checkpoint Blockade Induces Curative Anti-Tumor Immunity. Mol Ther Oncol (2018) 9:51–63. 10.1016/j.omto.2018.04.004 PMC6026467 29988525

[36] Scherwitzl I Opp S Hurtado AM Pampeno C Loomis C Kannan K. Sindbis Virus With Anti-OX40 Overcomes the Immunosuppressive Tumor Microenvironment of Low-Immunogenic Tumors. Mol Ther Oncol (2020) 17:431–47. 10.1016/j.omto.2020.04.012 PMC7251545 32478167

[37] Rice CM. Examples of Expression Systems Based on Animal RNA Viruses: Alphaviruses and Influenza Virus. Curr Opin Biotechnol (1992) 3:523–32. 10.1016/0958-1669(92)90081-S PMC7134803 1369402

[38] Bredenbeek PJ Frolov I Rice CM Schlesinger S. Sindbis Virus Expression Vectors: Packaging of RNA Replicons by Using Defective Helper RNAs. J Virol (1993) 67:6439–46. 10.1128/jvi.67.11.6439-6446.1993 PMC238079 8411346

[39] Langereis MA Stachura K Miller S Bosco-Lauth AM Albulescu IC Hartwig AE. An Alphavirus Replicon-Based Vaccine Expressing a Stabilized Spike Antigen Induces Sterile Immunity and Prevents Transmission of SARS-CoV-2 Between Cats. bioRxiv (2021) 2021.04.01.436305. 10.1101/2021.04.01.436305 PMC8528862 34671047

[40] Pastorino B Baronti C Gould EA Charrel RN de Lamballerie X. Effect of Chemical Stabilizers on the Thermostability and Infectivity of a Representative Panel of Freeze Dried Viruses. PloS One (2015) 10:e0118963. 10.1371/journal.pone.0118963 25923434 PMC4414529

[41] Tseng JC Hurtado A Yee H Levin B Boivin C Benet M. Using Sindbis Viral Vectors for Specific Detection and Suppression of Advanced Ovarian Cancer in Animal Models. Cancer Res (2004) 64:6684–92. 10.1158/0008-5472.CAN-04-1924 15374985

[42] Granot T Venticinque L Tseng JC Meruelo D. Activation of Cytotoxic and Regulatory Functions of NK Cells by Sindbis Viral Vectors. PloS One (2011) 6:e20598. 10.1371/journal.pone.0020598 21674047 PMC3107224

[43] Tseng JC Levin B Hirano T Yee H Pampeno C Meruelo D. *In Vivo* Antitumor Activity of Sindbis Viral Vectors. J Natl Cancer Inst (2002) 94:1790–802. 10.1093/jnci/94.23.1790 12464651

[44] Bell P Limberis M Gao G Wu D Bove MS Sanmiguel JC. An Optimized Protocol for Detection of E. Coli β-Galactosidase in Lung Tissue Following Gene Transfer. Histochem Cell Biol (2005) 124:77–85. 10.1007/s00418-005-0793-2 15947941

[45] Xia S Liu M Wang C Xu W Lan Q Feng S. Inhibition of SARS-CoV-2 (Previously 2019-Ncov) Infection by a Highly Potent Pan-Coronavirus Fusion Inhibitor Targeting its Spike Protein That Harbors a High Capacity to Mediate Membrane Fusion. Cell Res (2020) 30:343–55. 10.1038/s41422-020-0305-x PMC7104723 32231345

[46] Xia S Yan L Xu W Agrawal AS Algaissi A Tseng CK. A Pan-Coronavirus Fusion Inhibitor Targeting the HR1 Domain of Human Coronavirus Spike. Sci Adv (2019) 5:eaav4580. 10.1126/sciadv.aav4580 30989115 PMC6457931

[47] Lu L Liu Q Zhu Y Chan KH Qin L Li Y. Structure-Based Discovery of Middle East Respiratory Syndrome Coronavirus Fusion Inhibitor. Nat Commun (2014) 5:3067. 10.1038/ncomms4067 24473083 PMC7091805

[48] Scaglione A Patzig J Liang J Frawley R Bok J Mela A. PRMT5-Mediated Regulation of Developmental Myelination. Nat Commun (2018) 9:2840. 10.1038/s41467-018-04863-9 30026560 PMC6053423

[49] Dobin A Davis CA Schlesinger F Drenkow J Zaleski C Jha S. STAR: Ultrafast Universal RNA-Seq Aligner. Bioinf (Oxf Engl) (2013) 29:15–21. 10.1093/bioinformatics/bts635 PMC3530905 23104886

[50] Liao Y Smyth GK Shi W. The Subread Aligner: Fast, Accurate and Scalable Read Mapping by Seed-and-Vote. Nucleic Acids Res (2013) 41:e108. 10.1093/nar/gkt214 23558742 PMC3664803

[51] Love MI Huber W Anders S. Moderated Estimation of Fold Change and Dispersion for RNA-Seq Data With Deseq2. Genome Biol (2014) 15:550. 10.1186/s13059-014-0550-8 25516281 PMC4302049

[52] Subramanian A Tamayo P Mootha VK Mukherjee S Ebert BL Gillette MA. Gene Set Enrichment Analysis: A Knowledge-Based Approach for Interpreting Genome-Wide Expression Profiles. Proc Natl Acad Sci USA (2005) 102:15545–50. 10.1073/pnas.0506580102 PMC1239896 16199517

[53] Mootha VK Lindgren CM Eriksson K-F Subramanian A Sihag S Lehar J. PGC-1α-Responsive Genes Involved in Oxidative Phosphorylation are Coordinately Downregulated in Human Diabetes. Nat Genet (2003) 34:267–73. 10.1038/ng1180 12808457

[54] Szklarczyk D Gable AL Lyon D Junge A Wyder S Huerta-Cepas J. STRING V11: Protein-Protein Association Networks With Increased Coverage, Supporting Functional Discovery in Genome-Wide Experimental Datasets. Nucleic Acids Res (2019) 47:D607–13. 10.1093/nar/gky1131 PMC6323986 30476243

[55] Scharping NE Menk AV Moreci RS Whetstone RD Dadey RE Watkins SC. The Tumor Microenvironment Represses T Cell Mitochondrial Biogenesis to Drive Intratumoral T Cell Metabolic Insufficiency and Dysfunction. Immunity (2016) 45:701–3. 10.1016/j.immuni.2016.08.009 27653602

[56] Yu M Scherwitzl I Opp S Tsirigos A Meruelo D. Molecular and Metabolic Pathways Mediating Curative Treatment of a Non-Hodgkin B Cell Lymphoma by Sindbis Viral Vectors and Anti-4-1BB Monoclonal Antibody. J Immunother Cancer (2019) 7:185. 10.1186/s40425-019-0664-3 31307539 PMC6632218

[57] Blanco-Melo D Nilsson-Payant BE Liu W-C Møller R Panis M Sachs D. Imbalanced Host Response to SARS-CoV-2 Drives Development of COVID-19. Cell (2020) 181(5):1036–45.e9. 10.1016/j.cell.2020.04.026 PMC7227586 32416070

[58] Jackson LA Anderson EJ Rouphael NG Roberts PC Makhene M Coler RN. An mRNA Vaccine Against SARS-CoV-2 - Preliminary Report. N Engl J Med (2020) 383:1920–31. 10.1056/NEJMoa2022483 PMC7377258 32663912

[59] Corbett KS Flynn B Foulds KE Francica JR Boyoglu-Barnum S Werner AP. Evaluation of the mRNA-1273 Vaccine Against SARS-CoV-2 in Nonhuman Primates. N Engl J Med (2020) 383:1544–55. 10.1056/NEJMoa2024671 PMC7449230 32722908

[60] Sahin U Muik A Derhovanessian E Vogler I Kranz LM Vormehr M. COVID-19 Vaccine BNT162b1 Elicits Human Antibody and TH1 T Cell Responses. Nature (2020) 586:594–9. 10.1038/s41586-020-2814-7 32998157

[61] Zhou P Yang X-L Wang X-G Hu B Zhang L Zhang W. A Pneumonia Outbreak Associated With a New Coronavirus of Probable Bat Origin. Nature (2020) 579:270–3. 10.1038/s41586-020-2012-7 PMC7095418 32015507

[62] Ou X Liu Y Lei X Li P Mi D Ren L. Characterization of Spike Glycoprotein of SARS-CoV-2 on Virus Entry and its Immune Cross-Reactivity With SARS-CoV. Nat Commun (2020) 11:1620. 10.1038/s41467-020-15562-9 32221306 PMC7100515

[63] Gaebler C Wang Z Lorenzi JCC Muecksch F Finkin S Tokuyama M. Evolution of Antibody Immunity to SARS-CoV-2. Nature (2021) 591:639–44. 10.1038/s41586-021-03207-w PMC8221082 33461210

[64] Robbiani DF Gaebler C Muecksch F Lorenzi JCC Wang Z Cho A. Convergent Antibody Responses to SARS-CoV-2 in Convalescent Individuals. Nature (2020) 584:437–42. 10.1038/s41586-020-2456-9 PMC7442695 32555388

[65] Schmidt F Weisblum Y Muecksch F Hoffmann HH Michailidis E Lorenzi JCC. Measuring SARS-CoV-2 Neutralizing Antibody Activity Using Pseudotyped and Chimeric Viruses. J Exp Med (2020) 217:e20201181. 10.1084/jem.20201181 32692348 PMC7372514

[66] Bao L Deng W Huang B Gao H Liu J Ren L. The Pathogenicity of SARS-CoV-2 in Hace2 Transgenic Mice. Nature (2020) 583:830–3. 10.1101/2020.02.07.939389 32380511

[67] Rydyznski Moderbacher C Ramirez SI Dan JM Grifoni A Hastie KM Weiskopf D. Antigen-Specific Adaptive Immunity to SARS-CoV-2 in Acute COVID-19 and Associations With Age and Disease Severity. Cell (2020) 183:996–1012.e19. 10.1016/j.cell.2020.09.038 33010815 PMC7494270

[68] van der Windt GJ Everts B Chang CH Curtis JD Freitas TC Amiel E. Mitochondrial Respiratory Capacity is a Critical Regulator of CD8+ T Cell Memory Development. Immunity (2012) 36:68–78. 10.1016/j.immuni.2011.12.007 22206904 PMC3269311

[69] Li S Sullivan NL Rouphael N Yu T Banton S Maddur MS. Metabolic Phenotypes of Response to Vaccination in Humans. Cell (2017) 169:862–77.e17. 10.1016/j.cell.2017.04.026 28502771 PMC5711477

[70] Menk AV Scharping NE Moreci RS Zeng X Guy C Salvatore S. Early TCR Signaling Induces Rapid Aerobic Glycolysis Enabling Distinct Acute T Cell Effector Functions. Cell Rep (2018) 22:1509–21. 10.1016/j.celrep.2018.01.040 PMC5973810 29425506

[71] Jones N Cronin JG Dolton G Panetti S Schauenburg AJ Galloway SAE. Metabolic Adaptation of Human CD4+ and CD8+ T-Cells to T-Cell Receptor-Mediated Stimulation. Front Immunol (2017) 8:1516. 10.3389/fimmu.2017.01516 29170670 PMC5684100

[72] McElhaney JE Xie D Hager WD Barry MB Wang Y Kleppinger A. T Cell Responses are Better Correlates of Vaccine Protection in the Elderly. J Immunol (2006) 176:6333–9. 10.4049/jimmunol.176.10.6333 16670345

[73] McElhaney JE Ewen C Zhou X Kane KP Xie D Hager WD. Correlates With Protection and Enhanced CTL Response to Influenza Vaccination in Older Adults. Vaccine (2009) 27:2418–25. 10.1016/j.vaccine.2009.01.136 PMC2800816 19368783

[74] Hashimoto K Kouno T Ikawa T Hayatsu N Miyajima Y Yabukami H. Single-Cell Transcriptomics Reveals Expansion of Cytotoxic CD4 T Cells in Supercentenarians. Proc Natl Acad Sci (2019) 116:24242–51. 10.1073/pnas.1907883116 PMC6883788 31719197

[75] Folegatti PM Ewer KJ Aley PK Angus B Becker S Belij-Rammerstorfer S. Safety and Immunogenicity of the ChAdOx1 Ncov-19 Vaccine Against SARS-CoV-2: A Preliminary Report of a Phase 1/2, Single-Blind, Randomised Controlled Trial. Lancet (2020) 396:467–78. 10.1016/S0140-6736(20)31604-4 PMC7445431 32702298

[76] Erasmus JH Khandhar AP O’Connor MA Walls AC Hemann EA Murapa P. An Alphavirus-Derived Replicon RNA Vaccine Induces SARS-CoV-2 Neutralizing Antibody and T Cell Responses in Mice and Nonhuman Primates. Sci Transl Med (2020) 12:3125. 10.1126/scitranslmed.abc9396 PMC7402629 32690628

[77] Szurgot I Hanke L Sheward DJ Vidakovics LP Murrell B McInerney GM. DNA-Launched RNA Replicon Vaccines Induce Potent Anti-SARS-CoV-2 Immune Responses in Mice. Sci Rep (2021) 11:3125. 10.1038/s41598-021-82498-5 33542325 PMC7862230

[78] Henning L Endt K Steigerwald R Anderson M Volkmann A Monovalent A. And Trivalent MVA-Based Vaccine Completely Protects Mice Against Lethal Venezuelan, Western, and Eastern Equine Encephalitis Virus Aerosol Challenge. Front Immunol (2020) 11:598847. 10.3389/fimmu.2020.598847 33542715 PMC7851092

[79] Davis NL West A Reap E MacDonald G Collier M Dryga S. Alphavirus Replicon Particles as Candidate HIV Vaccines. IUBMB Life (2002) 53:209–11. 10.1080/15216540212657 12120997

[80] Fleeton MN Chen M Berglund P Rhodes G Parker SE Murphy M. Self-Replicative RNA Vaccines Elicit Protection Against Influenza A Virus, Respiratory Syncytial Virus, and a Tickborne Encephalitis Virus. J Infect Dis (2001) 183:1395–8. 10.1086/319857 11294672

[81] Andersson C Liljeström P Ståhl S Power UF. Protection Against Respiratory Syncytial Virus (RSV) Elicited in Mice by Plasmid DNA Immunisation Encoding a Secreted RSV G Protein-Derived Antigen. FEMS Immunol Med Microbiol (2000) 29:247–53. 10.1111/j.1574-695X.2000.tb01530.x 11118904

[82] Roques P Ljungberg K Kümmerer BM Gosse L Dereuddre-Bosquet N Tchitchek N. Attenuated and Vectored Vaccines Protect Nonhuman Primates Against Chikungunya Virus. JCI Insight (2017) 2:e83527. 10.1172/jci.insight.83527 28352649 PMC5358498

[83] Wressnigg N Hochreiter R Zoihsl O Fritzer A Bézay N Klingler A. Single-Shot Live-Attenuated Chikungunya Vaccine in Healthy Adults: A Phase 1, Randomised Controlled Trial. Lancet Infect Dis (2020) 20:1193–203. 10.1016/S1473-3099(20)30238-3 32497524

[84] van de Wall S Ljungberg K Ip PP Boerma A Knudsen ML Nijman HW. Potent Therapeutic Efficacy of an Alphavirus Replicon DNA Vaccine Expressing Human Papilloma Virus E6 and E7 Antigens. Oncoimmunology (2018) 7:e1487913. 10.1080/2162402X.2018.1487913 30288352 PMC6169581

[85] Lundstrom K. Alphavirus Vectors for Vaccine Production and Gene Therapy. Expert Rev Vaccines (2003) 2:445–59. 10.1586/14760584.2.3.445 12903809

[86] Avogadri F Zappasodi R Yang A Budhu S Malandro N Hirschhorn-Cymerman D. Combination of Alphavirus Replicon Particle-Based Vaccination With Immunomodulatory Antibodies: Therapeutic Activity in the B16 Melanoma Mouse Model and Immune Correlates. Cancer Immunol Res (2014) 2:448–58. 10.1158/2326-6066.CIR-13-0220 PMC4406637 24795357

[87] Khalil SM Tonkin DR Snead AT Parks GD Johnston RE White LJ. An Alphavirus-Based Adjuvant Enhances Serum and Mucosal Antibodies, T Cells, and Protective Immunity to Influenza Virus in Neonatal Mice. J Virol (2014) 88:9182–96. 10.1128/JVI.00327-14 PMC4136293 24899195

[88] Plotkin SA. Correlates of Protection Induced by Vaccination. Clin Vaccine Immunol (2010) 17:1055–65. 10.1128/CVI.00131-10 PMC2897268 20463105

[89] Ewer KJ Barrett JR Belij-Rammerstorfer S Sharpe H Makinson R Morter R. T Cell and Antibody Responses Induced by a Single Dose of ChAdOx1 Ncov-19 (AZD1222) Vaccine in a Phase 1/2 Clinical Trial. Nat Med (2021) 27:270–8. 10.1038/s41591-020-01194-5 33335323

[90] Song A Tang X Harms KM Croft M. OX40 and Bcl-xL Promote the Persistence of CD8 T Cells to Recall Tumor-Associated Antigen. J Immunol (2005) 175:3534–41. 10.4049/jimmunol.175.6.3534 16148096

[91] Skibinski DAG Jones LA Zhu YO Xue LW Au B Lee B. Induction of Human T-Cell and Cytokine Responses Following Vaccination With a Novel Influenza Vaccine. Sci Rep (2018) 8:18007. 10.1038/s41598-018-36703-7 30573748 PMC6301966

[92] Otani N Shima M Ueda T Ichiki K Nakajima K Takesue Y. Evaluation of Influenza Vaccine-Immunogenicity in Cell-Mediated Immunity. Cell Immunol (2016) 310:165–9. 10.1016/j.cellimm.2016.09.005 27665371

[93] van Doremalen N Lambe T Spencer A Belij-Rammerstorfer S Purushotham JN Port JR. ChAdOx1 nCoV-19 Vaccine Prevents SARS-CoV-2 Pneumonia in Rhesus Macaques. Nature (2020) 586:578–82. 10.1038/s41586-020-2608-y PMC8436420 32731258

[94] Chandrashekar A Liu J Martinot AJ McMahan K Mercado NB Peter L. SARS-CoV-2 Infection Protects Against Rechallenge in Rhesus Macaques. Sci (New York NY) (2020) 369:812–7. 10.1126/science.abc4776 PMC7243369 32434946

[95] Sekine T Perez-Potti A Rivera-Ballesteros O Strålin K Gorin JB Olsson A. Robust T Cell Immunity in Convalescent Individuals With Asymptomatic or Mild COVID-19. Cell (2020) 183:158–68.e14. 10.1016/j.cell.2020.08.017 32979941 PMC7427556

[96] Weiskopf D Schmitz KS Raadsen MP Grifoni A Okba NMA Endeman H. Phenotype and Kinetics of SARS-CoV-2-Specific T Cells in COVID-19 Patients With Acute Respiratory Distress Syndrome. Sci Immunol (2020) 5:eabd2071. 10.1126/sciimmunol.abd2071 32591408 PMC7319493

[97] Ljungberg K Liljeström P. Self-Replicating Alphavirus RNA Vaccines. Expert Rev Vaccines (2015) 14:177–94. 10.1586/14760584.2015.965690 25269775

